# Selpercatinib combination with the mitochondria-targeted antioxidant MitoQ effectively suppresses *RET*–mutant thyroid cancer

**DOI:** 10.1038/s41698-024-00536-7

**Published:** 2024-02-20

**Authors:** Wenjing Chen, Sophie Dream, Pui-Yin Leung, Pui-Kei Wu, Stuart Wong, Jong-In Park

**Affiliations:** 1 Department of Biochemistry, Medical College of Wisconsin, Milwaukee, WI 53226 USA; 2 Department of Surgery, Medical College of Wisconsin, Milwaukee, WI 53226 USA; 3 Department of Medicine, Medical College of Wisconsin, Milwaukee, WI 53226 USA

**Keywords:** Targeted therapies, Thyroid cancer

## Abstract

Genetic alternation of *REarranged during Transfection* (*RET*) that leads to constitutive RET activation is a crucial etiological factor for thyroid cancer. RET is known to regulate mitochondrial processes, although the underlying molecular mechanisms remain unclear. We previously showed that the multi-kinase inhibitors vandetanib and cabozantinib increase the mitochondrial membrane potential (Δψ_m_) in *RET*-mutated thyroid tumor cells and that this effect can be exploited to increase mitochondrial enrichment of Δψ_m_-sensitive agents in the tumor cells. In this study, we hypothesized that the RET-selective inhibitor, selpercatinib, can increase Δψ_m_ and, subsequently, tumor cell uptake of the mitochondria-targeted ubiquinone (MitoQ) to the level to break the mitochondrial homeostasis and induce lethal responses in *RET*-mutated thyroid tumor cells. We show that selpercatinib significantly increased Δψ_m_, and its combination with MitoQ synergistically suppressed *RET*-mutated human thyroid tumor cells, which we validated using RET-targeted genetic approaches. Selpercatinib and MitoQ, in combination, also suppressed *CCDC6-RET* fusion cell line xenografts in mice and prolonged animal survival more effectively than single treatments of each agent. Moreover, we treated two patients with *CCDC6-RET* or *RET*^M918T^ thyroid cancer, who could not take selpercatinib at regular doses due to adverse effects, with a dose-reduced selpercatinib and MitoQ combination. In response to this combination therapy, both patients showed tumor reduction. The quality of life of one patient significantly improved over a year until the tumor relapsed. This combination of selpercatinib with MitoQ may have therapeutic potential for patients with *RET*-mutated tumors and intolerant to regular selpercatinib doses.

## Introduction

*RET* (REarranged during Transfection) is a proto-oncogene that encodes a cell surface receptor tyrosine kinase (RTK) and is subject to various genetic alternations^1^. For example, *RET* fusions, which result in ligand-independent dimerization and constitutive activation of RET^2–4^, have been identified in 10–20% of papillary thyroid cancer (PTC)^5–7^, 1–2% of non-small cell lung cancer^8–10^, and 0.2–1.6% of colorectal cancer^11–13^. Somatic and germline *RET* mutations leading to constitutive RET activation are also found in cancers, including multiple endocrine neoplasia type 2 A and 2B (MEN2A and MEN2B) syndromes and familial medullary thyroid carcinoma (MTC)^14,15^. Upon activation, RET promotes various cellular processes, including cell growth, proliferation, and survival, mainly through the mitogen-activated protein kinase/extracellular signal-regulated kinase (ERK), the phosphatidylinositol-3 kinase/protein kinase B (AKT)^16^, and the Janus kinase/signal transducer and activator of transcription pathways^2^.

*RET* mutation is an actionable therapeutic target. While vandetanib and cabozantinib have been approved by the Food and Drug Administration (FDA) for *RET*-mutant MTC and *RET* fusion-positive differentiated thyroid cancer^17–19^, these inhibitors are not RET-selective and ineffective against *RET*^V804^ gatekeeper mutations. Selpercatinib (RETEVMO™) and pralsetinib (GAVRETO™) are highly selective RET inhibitors designed to overcome the gatekeeper mutations^20,21^ and approved by the FDA in 2020 for *RET*-mutant thyroid and lung cancers^22–24^. Moreover, in September 2022, the FDA granted accelerated approval to selpercatinib for tumor-agnostic treatment of adult patients with locally advanced or metastatic solid *RET* tumors^25^. While generally very well tolerated, these inhibitors cause adverse effects in patients, including hypertension and hepatotoxicity as the most common adverse effects^23,24^. These adverse effects cause permanent discontinuation of selpercatinib in about 5%, dosage interruptions in about 42%, and dose reductions in about 31% of the patients^23^. Therefore, it is important to design a strategy to use the drug effectively while reducing its adverse effects.

We previously showed that low-dose vandetanib and cabozantinib can synergize with mitochondria-targeted agents to suppress MEN2A and MEN2B MTC cells^26^. Mechanistically, these inhibitors increased mitochondrial membrane potential (Δψ_m_) in the *RET* tumor cells, subsequently facilitating tumor cell uptake and retention of Δψ_m_-sensitive triphenyl phosphonium (TPP)-conjugated carboxy-proxyl (MitoCP) and ubiquinone (MitoQ). Consequent enrichment of MitoCP and MitoQ beyond tumor cell tolerance disrupted mitochondrial homeostasis, triggering tumor cell death^26,27^. The TPP moiety has been used as a vehicle for mitochondria targeting of different functional moieties^28,29^. Of note, while MitoQ is currently used as a dietary supplement due to its beneficial effects on mitochondrial bioenergetics^30,31^ and vascular function^32–34^, it also suppresses tumor cells derived from different tumors, including MTC^26^, melanoma^35^, breast cancer^36–38^, and pancreatic cancer^39^. This tumor suppressive effect is mainly attributed to higher MitoQ accumulation in tumor cells due to higher basal Δψ_m_ in tumor cells. This information suggests that MitoQ could be a safe agent for tumor suppression if its tumor cell enrichment is selectively facilitated.

In this study, we reasoned that Δψ_m_-inducing effects of vandetanib and cabozantinib are due to RET inhibition and hypothesized that a low dose of selpercatinib will also increase Δψ_m_ in *RET*-mutated tumor cells, priming the tumor cells to MitoQ sensitivity. We demonstrate that a combination of selpercatinib and MitoQ can effectively and synergistically suppress *RET-*mutated thyroid cancers.

## Results

### Selpercatinib increases Δψ_m_ in *CCDC6-RET* fusion mutated PTC cells

**Fig. 1:**
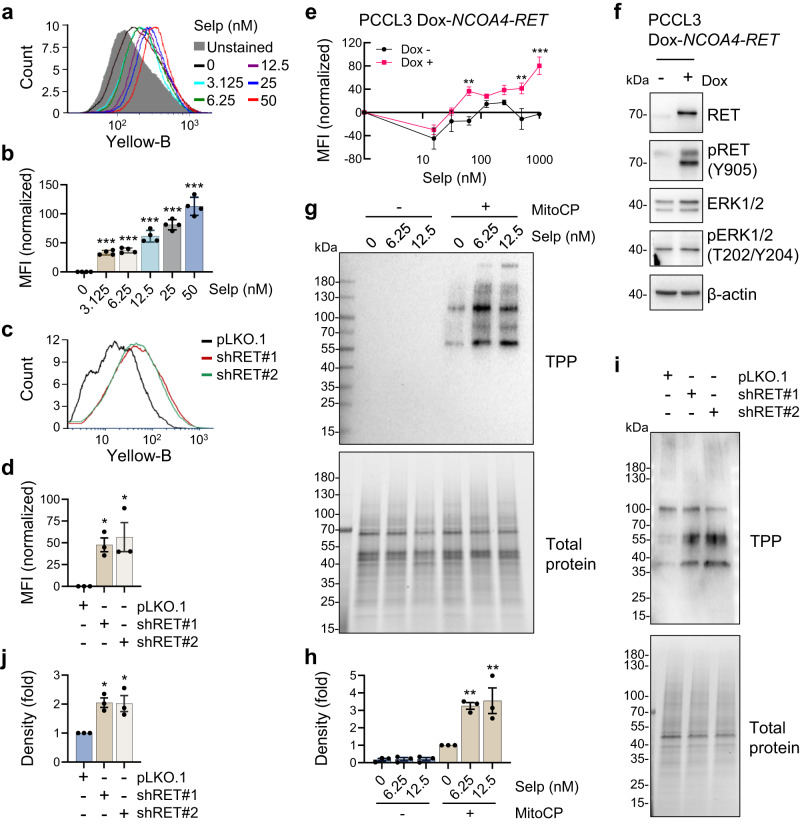
Selpercatinib alters mitochondrial membrane potential in cells expressing *RET* fusion. **a** TPC1 cells, treated with selpercatinib (selp) in a 3.125 to 50 nM dose range for 2 days, were stained with TMRM. Cellular TMRM retention was analyzed by flow cytometry measuring yellow fluorescence. **b** Mean fluorescence intensities (MFI) of TMRM-stained cells in **a** quantified by FCS Express software. Data are mean ± SEM (*N* = 4). ***P < 0.001 (compared with no treatment), one-way ANOVA with Bonferroni post-tests. **c** TPC1 cells, infected with lentiviral pLKO.1-shRET#1 and shRET#2 for 2 days, were stained with TMRM. Cellular TMRM retention was analyzed by flow cytometry measuring yellow fluorescence. **d** MFI of TMRM-stained cells in **c** quantified by FCS Express software. Data are mean ± SEM (*N* = 3). *P < 0.05 (compared with pLKO.1), one-way ANOVA with Bonferroni post-tests. **e** PCCL3 cells stably **e**xpressing a doxycycline (Dox)-inducible *NCOA4-RET* were treated with different doses of selpercatinib for 48 hours in the presence or absence of 0.5 µg/mL doxycycline. MFI of TMRM-stained cells was quantified by FCS Express software. Data are mean ± SEM (*N* = 4). **P < 0.005, ***P < 0.001 (compared with no doxycycline), two-way ANOVA with Bonferroni post-tests. **f** Western blot analysis of total lysates o**f** PCCL3 cells with or without 48-hour doxycycline treatment. **g** TPC1 cells pretreated with selpercatinib for 2 days were treated with 2 μM MitoCP for 1 hour. Mitochondrial lysates of these cells were analyzed by western blotting to detect the formation of TPP-adducts using an antibody specific to the TPP moiety of MitoCP. Total proteins were visualized in the stain-free gel as the control for equal protein loading. **h** Densitometry of the Western blot signals collected from three independent experiments performed as described in **g**. Signals were normalized for total protein intensity. Data are mean ± SEM (*N* = 3). **P < 0.005 (compared with no treatment), one-way ANOVA with Bonferroni post-tests. **i** Western blotting to detect TPP-adducts in the mitochondrial lysates of TPC1 cells, infected with pLKO.1-shRET#1 and shRET#2 for 2 days, and then treated with 2 μM MitoCP for 1 hour. Total proteins visualized in the stain-free gel are the control for equal protein loading. **j** Densitometry of the Western blot signals collected from three independent experiments performed as described in **i**. Data are mean ± SEM (*N* = 3). *P < 0.05 (compared with pLKO.1), one-way ANOVA with Bonferroni post-tests.

We determined whether selpercatinib can increase Δψ_m_ in TPC1 cells, the only human PTC cell line that carries a *coiled-coil domain containing 6 (CCDC6)-RET* fusion mutation, which is also known as *RET-PTC1*^40^. As determined by a dose-dependent analysis, we found that 72-hour treatment of selpercatinib between 1 nM and 1 µM dose ranges significantly decreased TPC1 cell viability with IC_50_ of 15 nM (Supplementary Fig. 1a). Western blot analysis of these cells revealed that selpercatinib substantially decreased RET phosphorylation at Tyr905, an activating autophosphorylation^16,41,42^, and phosphorylation of ERK1/2 and AKT, the key downstream effectors of RET^16^, while increasing expression of the cyclin-dependent kinase inhibitor p27^KIP1^ but decreasing p21^CIP1^ expression (Supplementary Fig. 1b, c). These effects are consistent with those of other RET inhibitors in this cell line^43,44^. Under these conditions validating selpercatinib potency, TPC1 cells stained with tetramethyl-rhodamine methyl ester (TMRM), a Δψ_m_-sensitive fluorescent dye, significantly increased in a selpercatinib dose-dependent manner (Fig. 1a, b). We confirmed that these selpercatinib effects are specific to the depletion of RET activity using RNA interference. Similar as selpercatinib, two short hairpin RNA (shRNA) constructs targeting different regions of *RET* mRNA consistently increased Δψ_m_ in TPC1 cells (Fig. 1c, d), while inducing growth inhibitory effects (Supplementary Fig. 1d–f). To further determine RET-specificity of this effect, we also used the immortalized normal rat thyrocytes PCCL3 expressing a doxycycline-inducible human *nuclear receptor coactivator 4* (*NCOA4*)*-RET*^45^, which is the second most common *RET* fusion in PTC^46^. Indeed, selpercatinib increased Δψ_m_ in this cell line model only upon doxycycline treatment (Fig. 1e, f). These data suggest that depletion of RET activity increases Δψ_m_ in *RET*-mutated PTC cells.

Next, we determined whether selpercatinib can sufficiently increase mitochondrial enrichment of a Δψ_m_-sensitive agent, using MitoCP as a tool compound. MitoCP contains TPP and the CP moiety, a 5-membered nitroxide free radical that can form a covalent conjugate with a thiol protein, which can be detected by a TPP-specific antibody^26,47^. For this, TPC1 cells pretreated with selpercatinib, were treated with MitoCP for 1 hour. When mitochondria fractions extracted from selpercatinib-pretreated cells were compared with the fractions extracted from unpretreated cells, much higher levels of TPP-protein adducts were detected by Western blotting in the cells pretreated with selpercatinib (Fig. 1g, h). Consistently, RET knockdown also increased TPP-protein adducts in TPC1 cells (Fig. 1i, j). These data suggest that selpercatinib can increase mitochondrial enrichment of a Δψ_m_-sensitive agent in *RET*-mutated tumor cells, similar as vandetanib and cabozantinib^26^.

### Selpercatinib and MitoQ can synergistically suppress the viability of *RET*-mutant PTC and MTC cells in culture

**Fig. 2:**
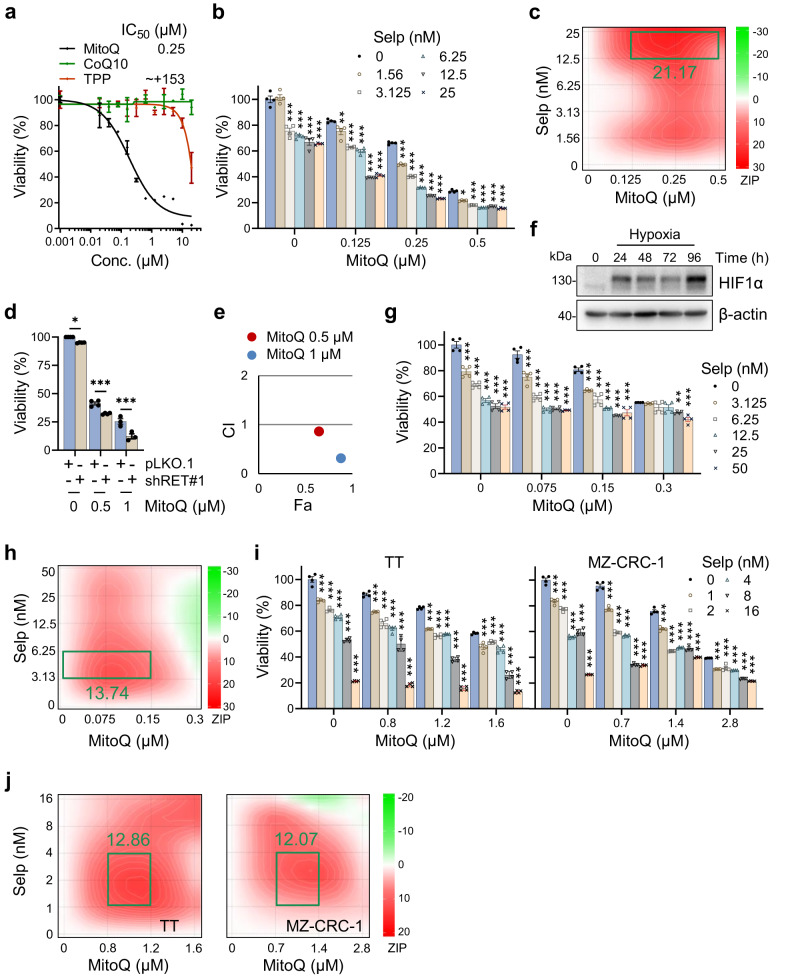
Selpercatinib synergizes with MitoQ to suppress the viability of *RET*-mutated thyroid tumor cells. **a** TPC1 cells in 12 well plates were treated with increasing doses of MitoQ, CoQ10, and TPP for 48 hours prior to crystal violet viability assay. Data are expressed as the percentage of untreated controls. Data are mean ± SEM (*N* = 3). **b** TPC1 cells, pretreated with different concentrations of selpercatinib, were treated with different doses of MitoQ for 48 hours prior to crystal violet viability assay. Data are mean ± SEM (*N* = 4). **c** SynergyFinder-generated plot of the viability data in **b**. The ZIP (zero interaction potency) score is indicated for the most synergistic area. **d** TPC1 cells, infected with lentiviral pLKO.1-shRET#1 for 24 hours, were treated with MitoQ for 48 hours prior to crystal violet viability assay. Data are expressed as the percentage of percentage of untreated controls. Data are mean ± SEM (*N* = 3). **e** Chou-Talalay plot of the data in **d**. CI was determined as a function of effect level (Fa). **f** Western blotting analysis of TPC1 cells cultured under hypoxia. β-actin is the control for equal protein loading. **g** TPC1 cells pretreated with selpercatinib were treated with MitoQ for 48 hours under the hypoxic condition prior to crystal violet viability assay. Data are mean ± SEM (*N* = 3). **h** SynergyFinder plot of the viability data in **g**. The ZIP score is indicated for the most synergistic area. **i** TT and MZ-CRC-1 cells pretreated with selpercatinib were treated with MitoQ for 48 hours prior to crystal violet viability assay. Data are mean ± SEM (*N* = 4). **j** SynergyFinder plot of the viability data in **i**. The ZIP score is indicated for the most synergistic area. All data are mean ± SEM (*N* ≥ 3). *P < 0.05, **P < 0.005, ***P < 0.001, Two-Way ANOVA with Bonferroni post-tests.

As the first step to evaluate the efficacy of MitoQ and its combination with selpercatinib in *RET*-mutant tumor cells, we validated that mitochondrial enrichment of its functional moiety, ubiquinone, is critical for its growth inhibitory effects in TPC1 cells. As determined by the crystal violet staining, MitoQ treatment for 48 hours significantly decreased TPC1 cell viability, exhibiting the IC_50_ value at 250 nM (Fig. 2a). In contrast, the functional moiety, CoQ10, did not significantly affect the cell viability while the vehicle, TPP, decreased cell viability only mildly at higher doses when these compounds were compared with MitoQ in identical dose ranges (Fig. 2a).

Having established the necessity of mitochondrial delivery of CoQ10 for suppressing TPC1 cell viability, we determined whether selpercatinib can synergize with MitoQ to suppress TPC1 cell viability in vitro. As determined by crystal violet staining, the combination of 24-hour selpercatinib pretreatment with a subsequent MitoQ treatment induced a robust viability loss in TPC1 cells (Fig. 2b). Of note, our analysis using SynergyFinder 2.0^48^ suggested that this viability loss is attributed to a synergy between these two agents (Fig. 2c and Supplementary Fig. 2). Consistent with this effect, a combination of RET knockdown and MitoQ treatment also effectively suppressed TPC1 cell viability (Fig. 2d), with a combination index (CI) suggesting synergy (Fig. 2e), as determined by Compusyn^49^. Importantly, tumors in clinical contexts are generally under hypoxic conditions^50^. We therefore determined whether the selpercatinib-MitoQ combination produces similar synergistic effects in TPC1 cells in Human Plasma-Like Medium under a hypoxic culture condition, which was validated by increased HIF1α expression (Fig. 2f). Indeed, selpercatinib and MitoQ synergistically suppressed the viability of TPC1 cells under this hypoxic condition (Fig. 2g, h), which is consistent with their effects under the normoxic culture condition.

These combinatory effects were not limited to TPC1 cells. The combination of 24-hour selpercatinib pretreatment with the subsequent MitoQ treatment also synergistically suppressed the viability of the human MTC cell lines, TT (*RET*^C634W^) and MZ-CRC-1 (*RET*^M918T^), in cultures, as determined by crystal violet staining (Fig. 2i, j; Supplementary Fig. 2). Consistently, RET knockdown also increased Δψ_m_ in these cells (Supplementary Fig. 3a, b). Moreover, overexpression of wild-type *RET*, *RET*^E632D/L633V/C634R^, and *RET*^M918T^ rendered HEK293 cells to increase Δψ_m_ upon selpercatinib treatment (Supplementary Fig. 3c, d), demonstrating that the selpercatinib on Δψ_m_ effect can be reconstituted in normal cells by increasing RET activity. These data strongly suggest that depletion of RET activity can synergize with MitoQ to suppress *RET*-mutant tumor cells.

### A combination of selpercatinib and MitoQ effectively suppresses the growth of TPC1 xenografts in mice

**Fig. 3:**
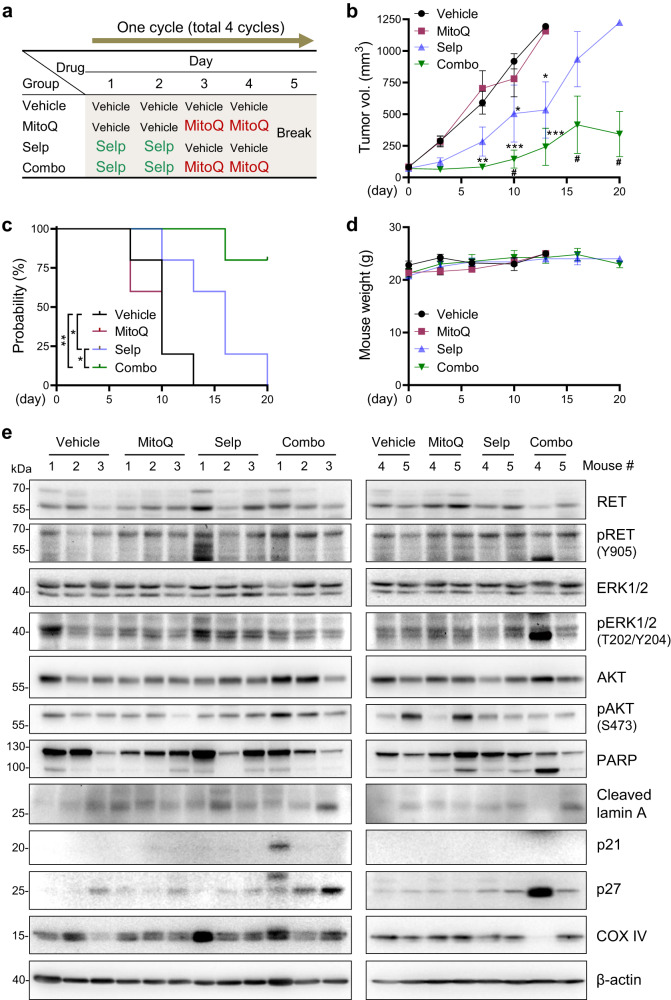
Selpercatinib and MitoQ combination effectively suppresses TPC1 xenografts in mice. **a** Treatment schedule for a cycle in total four cycles. Detailed information is in Materials and Method. **b** Changes in tumor sizes at the indicated time points (*N* = 5). *P < 0.05, **P < 0.005, ***P < 0.001 (relative to the vehicle); ^#^*P* < 0.05 (combination therapy versus selpercatinib monotherapy). Two-way ANOVA with Bonferroni post-tests. **c** Survival probability plot. Tumor size over 1200 mm^3^ was the endpoint. *P < 0.05, **P < 0.005, Log-rank test. **d** Body weights measured during the treatment. **e** Western blot analysis of tumor homogenates. β-actin is the control for equal protein loading.

We determined the preclinical efficacy of the selpercatinib and MitoQ combination using immune-compromised mice bearing TPC1 xenografts. These animals were subjected to 4 cycles of treatments wherein selpercatinib and MitoQ were orally administered singly or in combination. For the drug combination, each cycle consisted of 2-day selpercatinib treatment followed by 2-day MitoQ treatment and 1 drug holiday (Fig. 3a), which is similar to the schedule that we previously used for preclinical evaluation of the vandetanib and MitoCP combination [23]. For the comparison between mono- and combination therapies, we used selpercatinib at 0.5 mg/kg/dose, a much lower dose than the highly potent doses (16-30 mg/kg twice a day) in different preclinical models^51,52^, because its standard doses were too potent to determine the effect of its combination with other drugs (Supplementary Fig. 4). Likewise, we used MitoQ at 20 mg/kg/dose, which did not significantly reduce tumor volume in a preclinical breast cancer model albeit decreasing tumor metastasis^37^.

Consistent with the in vitro data above, the sequential administration of selpercatinib and MitoQ most effectively suppressed the growth of TPC1 xenografts when compared with the monotherapy groups (Fig. 3b), and significantly prolonged mouse survival (Fig. 3c) without affecting body weight (Fig. 3d). In contrast, the selpercatinib monotherapy group exhibited partial tumor suppression while the MitoQ monotherapy group did not show any difference from the control group. Although we also analyzed tumors by Western blotting, we could not detect any significant changes in the cleavage of poly (ADP-ribose) polymerase (PARP) and lamin A, expression of p27^KIP1^, p21^CIP1^, and cytochrome c oxidase subunit 4 (COX IV), and the activation phosphorylation of RET, ERK1/2, and AKT (Fig. 3e), which are the surrogate markers for cell death, cell cycle arrest, mitochondrial integrity, and RET signaling activity, respectively. These data suggest that the combination effect may be independent of these proteins, although the different timing of tumor harvest complicates the interpretation of these data.

### Patients with *RET-*mutant thyroid cancer tolerate and respond to combination therapy using selpercatinib and MitoQ

**Fig. 4:**
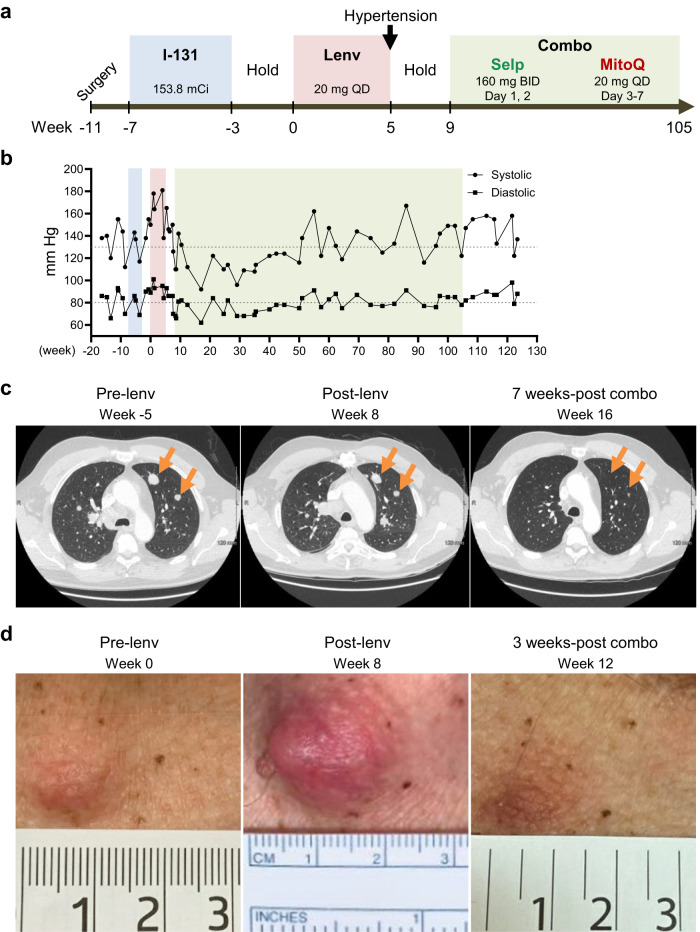
Treatment of the patient with *CCDC6-RET* fusion metastatic PTC. **a** Treatment timeline. **b** Blood pressure changes during the treatment. Dotted line indicates normal range of blood pressure. **c** CT scans of lung metastasis (orange arrows) prior to lenvatinib treatment (left), after stopping lenvatinib (middle), and after receiving 7 weeks of the selpercatinib and MitoQ combination therapy (right). Colors in **b** indicate the same treatment schedule shown in **a**. **d** Images of chest wall skin metastasis prior to lenvatinib treatment (left), after stopping lenvatinib (middle), and after receiving 3 weeks of the combination therapy (right).

We treated two patients with *RET*-mutant thyroid cancer with a selpercatinib and MitoQ combination. First patient was a 55-year-old man with poorly differentiated metastatic PTC with a history of poorly controlled hypertension. His tumor progressed after surgical removal of tumor mass and 153.8 mCi of I-131 for 4 weeks (Fig. 4a, full case report in the supplemental document). While awaiting approval for next-generation sequencing (NGS), the patient was treated with lenvatinib but this therapy was held after 5 weeks due to exacerbation of pre-existing refractory hypertension, grade 3, and symptoms of right heart failure (Fig. 4b). While on lenvatinib and during the hold, his cancer continued to progress and his heart dysfunction worsened (Fig. 4b, c). Because of the deteriorating medical status of this patient and the presence of *CCDC6-RET* fusion mutation in his tumor, we started a weekly cycled schedule for reduced selpercatinib dose with the addition of MitoQ: selpercatinib 160 mg twice a day (BID) on days 1, 2 (off days 3, 4, 5, 6, 7) followed by MitoQ 20 mg daily (QD) on days 3, 4, 5, 6, 7 (off days 1, 2) (Fig. 4a). The patient responded well to this treatment for 22 months, with a sustained radiographic partial response evaluated by response evaluation criteria in solid tumors (RECIST) following the first four months of treatment (Fig. 4c). A chest wall skin metastasis completely resolved during treatment (Fig. 4d). However, at 23 months of treatment, neck and chest CT scan revealed a new site of oligometastatic disease in the manubrium, which progressed over the next month.

**Fig. 5:**
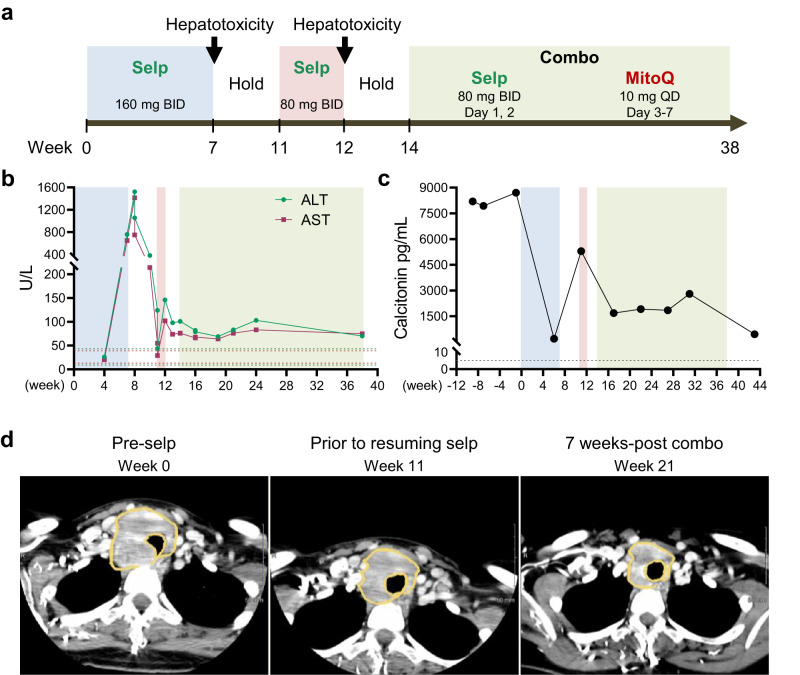
Treatment of the patient with locally advanced, unresectable *RET*^M918T^ MTC. **a** Treatment timeline. **b** Changes in ALT and AST. **c** Changes in serum calcitonin levels. Dotted lines in **b**, **c** indicate normal range of tests; colors indicate the same treatment schedule shown in **a**. **d** CT scans of right 7 cm MTC with mass effects on the trachea and esophagus (orange circle) prior to initiating selpercatinib treatment (left), prior to resuming reduced dosage of selpercatinib (middle), and after 7 weeks of the selpercatinib and MitoQ combination therapy (right).

The second patient was a 34-year-old female with *RET*^M918T^ locally advanced, unresectable MTC, exhibiting elevated carcinoembryonic antigen and calcitonin levels. This patient was initially started on selpercatinib 160 mg BID for 7 weeks (Fig. 5a, full case report in the supplemental document) but this treatment was on hold due to hepatotoxicity with elevated asymptomatic alanine transferase (ALT) and aspartate transferase (AST) (Fig. 5b). Selpercatinib was resumed in accordance with the FDA-approved package insert to dose level -2, 80 mg BID after liver function tests normalized (Fig. 5a). However, she briskly developed recurrent grade 2 transaminase elevation, nearly grade 3, soon after resumption of treatment (Fig. 5b). Therapy would normally be permanently discontinued after a second occurrence of grade 3/4 hepatotoxicity. However, due to her critical situation, selpercatinib was resumed in a reduced dose schedule with the addition of MitoQ using the following weekly cycled schedule: selpercatinib 80 mg BID on days 1, 2 (off days 3–7) followed by MitoQ 10 mg QD on days 3–7 (off days 1 and 2) (Fig. 5a). Calcitonin levels decreased after full dosage of selpercatinib, elevated again at reduced dose of selpercatinib, and decreased and stabilized during the combination therapy (Fig. 5c). Follow-up imaging after 7 weeks on this regimen indicated further reduction in tumor size with achievement of partial response by RECIST criteria (Fig. 5d). The treatment continued with the plateau of response on imaging at 24 weeks of treatment. Her treatment response and reduction in tumor size allowed her to undergo curative intent surgical resection.

## Discussion

**Fig. 6:**
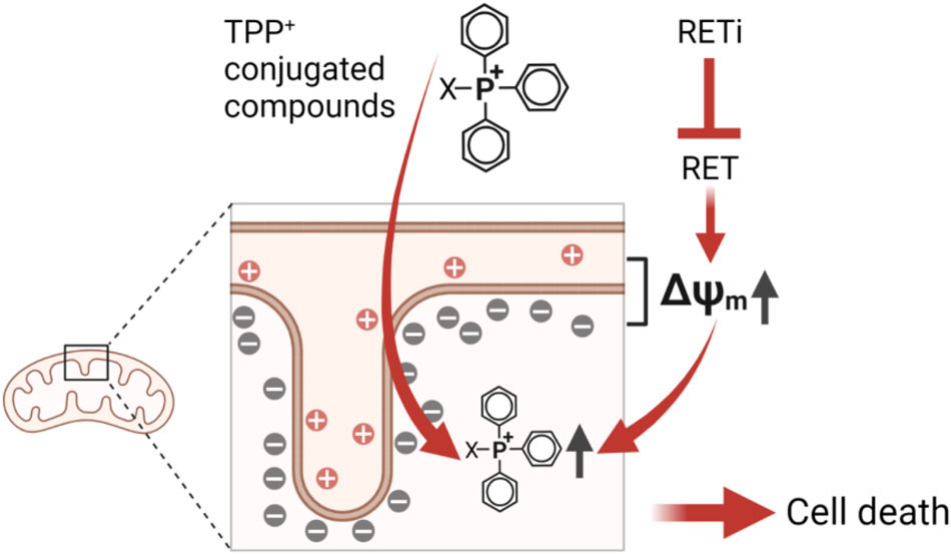
Graphic summary of the combination therapy concept. An increase in Δψ_m_ facilitates the mitochondrial accumulation of TPP cations. Because RET inhibition increases Δψ_m_, a RET inhibitor can enhance mitochondrial enrichment of a TPP-conjugated agent beyond a level to disrupt mitochondrial homeostasis and induce cell death. The image was generated with BioRender.com.

Previous studies demonstrated that many tumor cells, mainly derived from the cancers of thyroid, skin, and breast, are more sensitive to MitoQ than normal cells^35,36,38^. MitoQ sensitivity of these tumor cells is mainly attributed to the elevated steady-state Δψ_m_ of tumor cells, which is required to enhance their capacity to adapt to hypoxia, escape anoikis, and increase invasiveness^53,54^. Because the cationic TPP moiety of MitoQ is sensitive to Δψ_m_, TPP-conjugated agents would accumulate higher in the mitochondria of tumor cells than those of most normal cell types^34^, and the overenrichment of MitoQ would increase the probability of disrupting mitochondrial homeostasis. We thus reasoned that the property of RET inhibition to increase Δψ_m_ could be exploited to drive mitochondrial enrichment of MitoQ more effectively in tumor cells beyond the threshold to disrupt a bioenergetics balance (Fig. 6). Our data presented in this report strongly support this notion.

Selpercatinib and RET-specific RNA interference consistently increased Δψ_m_ in *CCDC6-RET* PTC cells, which supports RET-specificity of the selpercatinib effect. Together with our previous demonstration that vandetanib and cabozantinib can increase Δψ_m_ in MEN2A and MEN2B MTC cells^26^, these data suggest that RET is implicated in regulating mitochondrial activity in thyroid tumor cells. In support, *Ret* overexpression rescued muscle degeneration of the *Pink1* deficient Drosophila model of Parkinson’s disease (PD) by restoring the mitochondrial respiratory complex I activity partly through upregulating mRNA expression of its subunit, NDUFV2^55^. Of note, complex I inhibition induced mitochondrial hyperpolarization in HEK293 cells^56^, while a complex I defective mutation caused increased Δψ_m_ in neurons^57^. Similarly, knockout of NDUFS4, another complex I subunit, also increased Δψ_m_ in mouse embryonic fibroblasts^58^, although NDUFS4 knockout caused mitochondrial depolarization in mouse skeletal muscle cells^59^. These contrasting effects suggest that complex I activity can conditionally affect Δψ_m_, probably in a cell context-dependent manner as well as in a temporal- and magnitude-dependent manner. Indeed, short-term inhibition of the complex I by rotenone increased Δψ_m_ whereas its prolonged inhibition by the drug decreased Δψ_m_ in 143B cells^60^. Given this information, it remains to be answered whether different selpercatinib doses can induce differential effects on complex I activity.

By what mechanism does mitochondrial overenrichment of MitoQ induce tumor cell death? MitoQ is expected to function primarily as an antioxidant in cells. Tumor cells depend on reactive oxygen species (ROS) more than normal cells to facilitate tumor cell survival, angiogenesis, metastasis, and chemoresistance^61,62^. In this context, MitoQ would limit tumor cell survival by depleting ROS. Indeed, an oxidized form of ubiquinone (BPM31510) induces anti-cancer effects and is currently tested for solid tumors with high ROS burden in clinical trials^63^. An alternative scenario is also available. Although initially designed as an antioxidant, MitoQ can elevate ROS production in cells. Studies using mitochondria isolated from bovine aortic endothelial cells showed that MitoQ can increase ROS generation by mitochondrial complex I while mildly decreasing ROS generation by mitochondrial complex II without any effect on complex III^64,65^. In contrast, its functional moiety CoQ10 had no similar effects^64,65^. Mechanistically, MitoQ is poorly reduced by complex I because of the bulkiness of TPP moiety that sterically hinders the access of the ubiquinone group to the complex I^66,67^. However, TPP does not affect the access of ubiquinone moiety to the complex II, which makes MitoQ a good substrate for the complex II^67^. Meanwhile, MitoQ is not oxidized by Complex III^66^ because the positive charge of the TPP moiety limits the access of MitoQ to the active site(s) of complex III^67^. Of note, MitoQ treatment of endothelial cells increased superoxide production from malate- and glutamate-fueled complex I, leading to the onset of apoptosis^64^. Because many tumor types maintain a higher level of malate and glutamate level to fuel complex I^68^, MitoQ might have pro-oxidant effects in most metabolically active tumor cells. One may question that MitoQ sensitivity of certain normal cell types would limit the use of high dose MitoQ for cancer therapy and requires a strategy to selectively increase MitoQ delivery into tumor cells for effective treatment. We posit that selpercatinib preferentially sensitize *RET-*mutated tumor cells to MitoQ, proposing that our strategy to combine selpercatinib and MitoQ might confer an advantage in therapeutically using the agent.

We also appreciate the possibility that selpercatinib and MitoQ synergize through additional mechanisms. In a tubular injury mouse model and human kidney 2 (HK-2) cells, MitoQ partially attenuated mitophagic cell death caused by PINK/Parkin-deficiency by increasing Nrf2 level^69^. Similarly, MitoQ enhanced PINK/Parkin-mediated mitophagy to inhibit hepatic stellate cell activation and liver fibrosis^70^, and to ameliorate myocardial ischemia-reperfusion injury in type 2 diabetic rats^71^. Meanwhile, *Ret* overexpression rescued muscle degeneration of the *Pink1* deficient Drosophila PD model^55^, and functional redundancy between RET and Parkin in maintaining mitochondrial function and morphology has been demonstrated in a mouse PD model^72^. Given this information, MitoQ and RET might have effects that overlap in association with PINK. Therefore, it is also possible that RET inhibition and MitoQ may synergistically target a PINK/Parkin-mediated mechanism. Lastly, MitoQ may support the general health status of patients undergoing chemotherapy, as previously suggested^73,74^. Indeed, MitoQ improved muscle atrophy and weakness of the mice bearing colon cancer cell line xenografts^74^.

Clinically, the recommended dosing of selpercatinib for a body weight of ≥50 kg is 160 mg BID, while 120 mg BID for patients weighing <50 kg^23^. The adverse effects of selpercatinib result in permanent discontinuation or dose reduction in about 5% and 31% of patient, respectively^23^. The two patients in this report did not tolerate standard doses of selpercatinib due to adverse effects. However, these patients did tolerate reduced doses of selpercatinib when combined with MitoQ. Whether MitoQ significantly enhanced the effects of selpercatinib cannot be determined in this brief analysis of only two patients. Nevertheless, these observations suggest a strategy to effectively use selpercatinib in the patients with low tolerance to this drug. MitoQ has been confirmed for its safety in humans, and its clinical benefits are currently being evaluated in Phase II trials^32–34^, although its benefit for cancer patients has not yet been tested in the setting of a clinical trial. We believe our preclinical data and patient cases are pertinent to the design of a future clinical trial to determine whether selpercatinib and MitoQ combination can provide a clinical benefit to patients with *RET*-mutated cancers.

## Methods

### Cell culture and reagents

The human thyroid cancer cell line TPC1 (ATCC, Manassas, VA) was maintained in RPMI 1640 (Invitrogen, Thermo Fisher Scientific, Waltham, MA, #11875) supplemented with 10% heat-inactivated fetal bovine serum (FBS, Gibco, Thermo Fisher Scientific, #16000-044) and 100 U of Penicillin-Streptomycin (Gibco, #15140) per ml. TT was maintained in DMEM supplemented with 16% heat-inactivated FBS and 100 U of Penicillin-Streptomycin per ml. MZ-CRC-1 was maintained in DMEM (Gibco, #11965) supplemented with 10% heat-inactivated FBS and 100 U of Penicillin-Streptomycin per ml. As indicated in the text, cells were also maintained in a Human Plasma-Like Medium (HPLM, Gibco, #A4899101) supplemented with heat-inactivated 10% FBS, and 100 U Penicillin-Streptomycin per ml. HEK293T was maintained in DMEM supplemented with 10% BGS (Hyclone, Cytiva, Muskegon, MI, #SH30541.03). PCCL3 was maintained in Ham F12 media (MilliporeSigma, N6658), supplemented with 5% FBS, 1 mIU/mL bovine thyroid stimulating hormone (SCRIPPS, CA, T0117), 10 μg/mL insulin (Gibco, # 41400045), 5 μg/mL apo-transferrin (MilliporeSigma, T2252), 10 nM of hydrocortisone (MilliporeSigma, #H0888) and 100 U of Penicillin-Streptomycin. Hypoxic cell culture was carried out in a humidified incubator with 1% O_2_, 5% CO_2_, and 94% N_2_. All experiments were performed using cells within ten passages from the acquisition point. Selpercatinib was purchased from Chemietek (Indianapolis, IN, #CT-LX292). MitoCP was obtained from Dr. Balaraman Kalyanaraman (Biophysics, Medical College of Wisconsin). MitoQ ([10-(4,5-dimethoxy-2-methyl-3,6-dioxo-1,4-cyclohexadien-1-yl) decyl] triphenyl phosphonium) used for in vitro and preclinical studies was obtained from MitoQ (Auckland, New Zealand); MitoQ for patient treatments was purchased from Amazon.com. CoQ10 (#C9538) and methyl-TPP (#130079) were purchased from MilliporeSigma (St. Louis, MO).

### Cell viability and cell cycle analyses

IC_50_ values and combination effects of drug treatments were determined by Crystal Violet (Fisher Chemical, Thermo Fisher Scientific, #C58125) cell viability staining assay. Briefly, cells in 96 well-plates were fixed in formaldehyde (Fisher Chemical, #BP531), stained with 0.05% Crystal Violet for 30 minutes, washed with water three times, air-dried, and incubated in 200 µl of methanol (VWR Chemicals BDH^®^, Radnor, PA #BDH1135) for 10 min at room temperature before measuring absorbance at 540 nm. Cell viability for RET knockdown was determined by flow cytometry of cells stained with the fluorescent DNA intercalator Thiazole Red (TO-PRO^®^3, Invitrogen, # T3605) using Guava EasyCyte flow cytometry system (MilliporeSigma, #05005007). Data were analyzed by FCS EXPRESS software (De Novo Software, Los Angeles, California). For the cell cycle, flow cytometry of cells stained with propidium iodide (PI, Invitrogen, #P1304MP) was performed using the Guava EasyCyte flow cytometry system, as previously described^75^.

### Detection of Δψ_m_ using TMRM

Cells were incubated with culture medium containing 2 nM TMRM (Invitrogen, #T668) in 12-well plates for 30 min at 37 °C in dark, collected by trypsinization, resuspended in phosphate-buffered saline containing 0.5% bovine serum albumin, and analyzed using Guava EasyCyte flow cytometry system Data were analyzed by FCS EXPRESS software flow, as described previously^27^.

### Immunoblotting

Mitochondrial fractions were extracted using the Mitochondria Isolation Kit for Cultured Cells (Thermo Fisher Scientific, #89874). For total cell lysate, cells were harvested in a lysis buffer containing 62.5 mM Tris-HCl (pH 6.8), 2% sodium dodecyl sulfate (SDS), and the protease and phosphatase inhibitor cocktails 2 and 3 (MilliporeSigma, #P8340, P5726, and P0044, respectively). Protein concentration was determined using the Pierce™ BCA Protein Assay Kit (Pierce, Thermo Fisher Scientific, #23227). Protein samples were resolved on the SDS-polyacrylamide gel electrophoresis and transferred to the polyvinylidene difluoride membrane filter (Bio-Rad, Hercules, CA, #1620177). After transfer, membranes were blocked in a buffer containing 0.1 M Tris (pH 7.4), 0.9% NaCl, 0.1% Tween 20, and 5% nonfat dry milk for 1 h at 25 °C. Membranes were then incubated with an appropriate antibody overnight at 4 °C with agitation at the following dilutions: RET (Cell Signaling, Danvers, MA, #3223) 1:2000; phospho-RET Try905 (Cell Signaling, #3221) 1:1000; ERK (Cell Signaling, #9102) 1:2000; phospho-ERK Thr202/Tyr204 (Santa Cruz Biotechnology, Santa Cruz, CA, #sc-16982) 1:1000; AKT (Cell Signaling, #9272) 1:2000; phospho-AKT Ser473 (Cell Signaling, #9271); PARP (Cell Signaling, #9542) 1:2000; cleaved lamin A (Cell Signaling, #2035) 1:1000; cytochrome c oxidase subunit IV (COX IV, Cell Signaling, #4850) 1:2000; p21^CIP1^ (Santa Cruz Biotechnology, #sc-56335) 1:1000, p27^KIP1^ (Santa Cruz Biotechnology, #sc-1641) 1:1000; HIF1α (Cell Signaling, #14179) 1:1000 and β-actin (MilliporeSigma, #A2228) 1:5000. Chemiluminescence signals of immunoblots were visualized by the chemiluminescence kits SuperSignal West Pico (#34079, Pierce) and Femto (#23227, Pierce), captured by ChemiDoc XRS+ (Bio-Rad, Hercules, CA), and analyzed by Image Lab software (Bio-Rad) for densitometry. All blots were derived from the same experiment and were processed in parallel. Their original uncropped images are provided in Supplementary Fig. 5.

### RNA interference and recombinant lentiviral constructs

RET was depleted using two independent lentiviral shRNA expression systems, pLKO-shRET#1 (MilliporeSigma, #NM_000323) and pLKO-shRET#2 (#NM_020629), which target CCGCTGGTGGACTGTAATAAT and CCTCATCTCATTTGCCTGGCA in human *RET*, respectively. Lentiviral pHAGE expressing human wild-type *RET*, *RET*^E632D/L633V/C634R^, and *RET*^M918T^ were generated by ligating each *RET* encoding gene into the NheI/SphI site of the pHAGE-GFP vector^76^. Lentiviruses were generated from HEK293T cells and used, as we previously described^76^.

### Tumor xenografts

A total of 5 × 10^6^ TPC1 cells suspended in 100 µL of Hank’s balanced salt solution (Gibco, #14025) mixed with Extracellular Matrix Gel (MilliporeSigma, #E6909) at 1:1 ratio was inoculated subcutaneously into the rear flanks of 6-week-old female athymic nude (*nu*/*nu*) mice (The Jackson Laboratory, Bar Harbour, ME, #007850). Once palpable, tumors were measured using Vernier calipers twice a week. Tumor volumes were calculated using the formula: length × width × height × 0.5236. When tumor volumes reached 50 mm^3^, mice were randomized and sorted into 4 groups of 5 animals to achieve equal tumor size distribution in all treatment groups. Mice were treated with vehicle, MitoQ, selpercatinib, and the combination of two compounds, respectively. Drugs dissolved in 100 μl vehicle (1:12 mixture of DMSO/15% β-cyclodextrin) were orally administered by gavage daily for 4 days, followed by 1-day break. Four cycles of this treatment were conducted. The control group received only the vehicle, the MitoQ group received 20 mg drug/kg body weight/dose, the selpercatinib group received 0.5 mg drug/kg body weight/dose, and the combination group received two doses of selpercatinib followed by two doses of MitoQ in a cycle. Tumor sizes were measured, and survival probability was also plotted. Ethical endpoints were when tumor size reached 1200 mm^3^. At the end of the experiments, animals were euthanized by CO_2_ asphyxiation, and tumor tissues were harvested. All animal studies were performed according to protocols approved by the Institutional Animal Care and Use Committee at the Medical College of Wisconsin (AUA00001327).

### Patients

The patients were treated at the Froedtert Hospital and Medical College of Wisconsin. Analysis and reporting of patients’ treatment and outcome were performed in accordance with the Declaration of Helsinki under an Institutional Review Board-approved study (IRB number PRO00002787) that waives the requirement for informed consent because of the use of de-identified data. Responses were assessed by RECIST 1.1^77^. Adverse events were graded using the Common Terminology Criteria for Adverse Events version 4.03^78^. NGS revealed *CCDC6-RET* fusion; *CDKN2B* loss; *CDKN2A* loss; *TERT* promoter^-124C>T^; *TP53*^R248Q^ in the tumor of the first patient, which was performed by Linda T. and John A. Mellowes Center for Genomic Sciences and Precision Medicine (Medical College of Wisconsin, Wisconsin, WI) and FoundationOne^®^ CDx (Foundation Medicine, Cambridge, MA); and *RET*^M918T^ in the tumor of the second patient, which was performed by TEMPUS (Chicago, IL).

### Quantification and statistical analyses

All graphs represent the mean ± the standard deviation of biological triplicates’ mean. Measurements were taken from distinct samples. Statistical significance was determined by one-way or two-way ANOVA with Bonferroni post-tests and two-tailed unpaired Student’s *t* test using PRISM (Graph-Pad Software, La Jolla, CA). IC_50_ was determined by PRISM. *P* values of the Kaplan–Meier curves were determined by the Log-rank test by PRISM. *P* values of <0.05 were considered statistically significant.

### Reporting summary

Further information on research design is available in the Nature Research Reporting Summary linked to this article.

### Supplementary information

Reporting Summary

Supplementary figures and patient case info

## Data Availability

All data needed to evaluate the conclusions in the paper are present in the paper and the Supplementary Materials. Any additional information required to reanalyze the data reported in this paper is available from the corresponding authors upon request. Reagents and materials produced in this study are available pending a completed Materials Transfer Agreement. The raw NGS data for the genomic analysis of patient tumors are not publicly available to protect patient privacy, but qualified researchers may apply for access to the datasets via a collaboration or data usage agreement.

## Code availability

This paper does not report any original code.

## References

[1] Santoro M, Carlomagno F (2013). Central role of RET in thyroid cancer. Cold Spring Harb. Perspect. Biol..

[2] Qian Y (2014). KIF5B-RET fusion kinase promotes cell growth by multilevel activation of STAT3 in lung cancer. Mol. Cancer.

[3] Bongarzone I (1993). Molecular characterization of a thyroid tumor-specific transforming sequence formed by the fusion of ret tyrosine kinase and the regulatory subunit RI alpha of cyclic AMP-dependent protein kinase A. Mol. Cell Biol..

[4] Tong Q, Xing S, Jhiang SM (1997). Leucine zipper-mediated dimerization is essential for the PTC1 oncogenic activity. J. Biol. Chem..

[5] Grieco M (1990). PTC is a novel rearranged form of the ret proto-oncogene and is frequently detected in vivo in human thyroid papillary carcinomas. Cell.

[6] Eszlinger M (2022). Systematic population-based identification of NTRK and RET fusion-positive thyroid cancers. Eur. Thyroid J..

[7] Pekova B (2020). RET, NTRK, ALK, BRAF, and MET fusions in a large cohort of pediatric papillary thyroid carcinomas. Thyroid.

[8] Kohno T (2012). KIF5B-RET fusions in lung adenocarcinoma. Nat. Med..

[9] Takeuchi K (2012). RET, ROS1 and ALK fusions in lung cancer. Nat. Med..

[10] Lipson D (2012). Identification of new ALK and RET gene fusions from colorectal and lung cancer biopsies. Nat. Med..

[11] Pietrantonio F (2018). RET fusions in a small subset of advanced colorectal cancers at risk of being neglected. Ann. Oncol..

[12] Kim SY (2018). NCOA4-RET fusion in colorectal cancer: therapeutic challenge using patient-derived tumor cell lines. J. Cancer.

[13] Le Rolle AF (2015). Identification and characterization of RET fusions in advanced colorectal cancer. Oncotarget.

[14] Salvatore D (2001). Increased in vivo phosphorylation of ret tyrosine 1062 is a potential pathogenetic mechanism of multiple endocrine neoplasia type 2B. Cancer Res..

[15] Santoro M (1995). Activation of RET as a dominant transforming gene by germline mutations of MEN2A and MEN2B. Science.

[16] Raman R (2022). Inhibition of FGF receptor blocks adaptive resistance to RET inhibition in CCDC6-RET-rearranged thyroid cancer. J. Exp. Med..

[17] Cabanillas ME, Brose MS, Holland J, Ferguson KC, Sherman SI (2014). A phase I study of cabozantinib (XL184) in patients with differentiated thyroid cancer. Thyroid.

[18] Degrauwe N, Sosa JA, Roman S, Deshpande HA (2012). Vandetanib for the treatment of metastatic medullary thyroid cancer. Clin. Med. Insights Oncol..

[19] Nagilla M, Brown RL, Cohen EE (2012). Cabozantinib for the treatment of advanced medullary thyroid cancer. Adv. Ther..

[20] Subbiah V (2021). Structural basis of acquired resistance to selpercatinib and pralsetinib mediated by non-gatekeeper RET mutations. Ann. Oncol..

[21] Wirth LJ (2019). Emergence and targeting of acquired and hereditary resistance to multikinase RET inhibition in patients with RET-altered cancer. JCO Precis. Oncol..

[22] Markham A (2020). Selpercatinib: first approval. Drugs.

[23] Bradford D (2021). FDA approval summary: selpercatinib for the treatment of lung and thyroid cancers with RET gene mutations or fusions. Clin Cancer Res..

[24] Kim J (2021). FDA approval summary: pralsetinib for the treatment of lung and thyroid cancers with RET gene mutations or fusions. Clin. Cancer Res..

[25] FDA. FDA grants accelerated approval to dabrafenib in combination with trametinib for unresectable or metastatic solid tumors with BRAF V600E mutation. (2022).

[26] Starenki D, Hong SK, Wu PK, Park JI (2017). Vandetanib and cabozantinib potentiate mitochondria-targeted agents to suppress medullary thyroid carcinoma cells. Cancer. Biol. Ther..

[27] Starenki D, Park JI (2013). Mitochondria-targeted nitroxide, Mito-CP, suppresses medullary thyroid carcinoma cell survival in vitro and in vivo. J. Clin. Endocrinol. Metab..

[28] Kulkarni CA (2021). A novel triphenylphosphonium carrier to target mitochondria without uncoupling oxidative phosphorylation. J. Med. Chem..

[29] Zielonka J (2017). Mitochondria-targeted triphenylphosphonium-based compounds: syntheses, mechanisms of action, and therapeutic and diagnostic applications. Chem. Rev..

[30] Kelso GF (2001). Selective targeting of a redox-active ubiquinone to mitochondria within cells: antioxidant and antiapoptotic properties. J. Biol. Chem..

[31] Murphy MP, Smith RA (2007). Targeting antioxidants to mitochondria by conjugation to lipophilic cations. Annu. Rev. Pharmacol. Toxicol..

[32] Park SY (2020). Acute mitochondrial antioxidant intake improves endothelial function, antioxidant enzyme activity, and exercise tolerance in patients with peripheral artery disease. Am. J. Physiol. Heart Circ. Physiol..

[33] Rossman MJ (2018). Chronic supplementation with a mitochondrial antioxidant (MitoQ) improves vascular function in healthy older adults. Hypertension.

[34] Smith RA, Murphy MP (2010). Animal and human studies with the mitochondria-targeted antioxidant MitoQ. Ann. N Y Acad. Sci..

[35] Hong SK, Starenki D, Wu PK, Park JI (2017). Suppression of B-Raf(V600E) melanoma cell survival by targeting mitochondria using triphenyl-phosphonium-conjugated nitroxide or ubiquinone. Cancer Biol. Ther..

[36] Capeloa T (2022). MitoQ inhibits human breast cancer cell migration, invasion and clonogenicity. Cancers (Basel).

[37] Capeloa T (2022). MitoQ prevents human breast cancer recurrence and lung metastasis in mice. Cancers (Basel).

[38] Rao VA (2010). The antioxidant transcription factor Nrf2 negatively regulates autophagy and growth arrest induced by the anticancer redox agent mitoquinone. J. Biol. Chem..

[39] Capeloa T (2022). Inhibition of mitochondrial redox signaling with mitoq prevents metastasis of human pancreatic cancer in mice. Cancers (Basel).

[40] Ishizaka Y (1989). Presence of aberrant transcripts of ret proto-oncogene in a human papillary thyroid carcinoma cell line. Jpn. J. Cancer Res..

[41] Iwashita T (1999). Biological and biochemical properties of Ret with kinase domain mutations identified in multiple endocrine neoplasia type 2B and familial medullary thyroid carcinoma. Oncogene.

[42] Hu X, Liu X, Khatri U, Wu J (2023). The heterogeneous transition state of resistance to RET kinase inhibitors converges on ERK1/2-driven Aurora A/B kinases. Drug Resist. Updat..

[43] Mologni L (2006). Inhibition of RET tyrosine kinase by SU5416. J. Mol. Endocrinol..

[44] Vitagliano D (2004). Regulation of p27Kip1 protein levels contributes to mitogenic effects of the RET/PTC kinase in thyroid carcinoma cells. Cancer Res..

[45] Knauf JA, Kuroda H, Basu S, Fagin JA (2003). RET/PTC-induced dedifferentiation of thyroid cells is mediated through Y1062 signaling through SHC-RAS-MAP kinase. Oncogene.

[46] Nikiforov YE (2002). RET/PTC rearrangement in thyroid tumors. Endocr. Pathol..

[47] Lin TK (2002). Specific modification of mitochondrial protein thiols in response to oxidative stress: a proteomics approach. J. Biol. Chem..

[48] Ianevski A, Giri AK, Aittokallio T (2020). SynergyFinder 2.0: visual analytics of multi-drug combination synergies. Nucleic Acids Res..

[49] Chou T, M. N. CompuSyn software. CompuSyn for drug combinations: PC software and user’s guide: a computer program for quantitation of synergism and antagonism in drug combinations, and the determination of IC50 and ED50 and LD50 values. ComboSyn Inc., Paramus, NJ. (2005).

[50] Hompland T, Fjeldbo CS, Lyng H (2021). Tumor hypoxia as a barrier in cancer therapy: why levels matter. Cancers (Basel).

[51] Shen T (2021). The L730V/I RET roof mutations display different activities toward pralsetinib and selpercatinib. NPJ Precis. Oncol..

[52] Subbiah V (2018). Selective RET kinase inhibition for patients with RET-altered cancers. Ann. Oncol..

[53] Mani S, Swargiary G, Singh KK (2020). Natural agents targeting mitochondria in cancer. Int. J. Mol. Sci..

[54] Heerdt BG, Houston MA, Augenlicht LH (2006). Growth properties of colonic tumor cells are a function of the intrinsic mitochondrial membrane potential. Cancer Res..

[55] Klein P (2014). Ret rescues mitochondrial morphology and muscle degeneration of Drosophila Pink1 mutants. EMBO J.

[56] Forkink M (2014). Mitochondrial hyperpolarization during chronic complex I inhibition is sustained by low activity of complex II, III, IV and V. Biochim. Biophys. Acta.

[57] Abramov AY (2010). Mechanism of neurodegeneration of neurons with mitochondrial DNA mutations. Brain.

[58] Valsecchi F (2012). Metabolic consequences of NDUFS4 gene deletion in immortalized mouse embryonic fibroblasts. Biochim. Biophys. Acta.

[59] Alam MT (2015). Skeletal muscle mitochondria of NDUFS4-/- mice display normal maximal pyruvate oxidation and ATP production. Biochim. Biophys. Acta.

[60] Barrientos A, Moraes CT (1999). Titrating the effects of mitochondrial complex I impairment in the cell physiology. J. Biol. Chem..

[61] Szatrowski TP, Nathan CF (1991). Production of large amounts of hydrogen peroxide by human tumor cells. Cancer Res..

[62] Galadari S, Rahman A, Pallichankandy S, Thayyullathil F (2017). Reactive oxygen species and cancer paradox: to promote or to suppress?. Free Radic. Biol. Med..

[63] Dadali T (2021). Elevated levels of mitochondrial CoQ(10) induce ROS-mediated apoptosis in pancreatic cancer. Sci. Rep..

[64] Doughan AK, Dikalov SI (2007). Mitochondrial redox cycling of mitoquinone leads to superoxide production and cellular apoptosis. Antioxid. Redox Signal..

[65] O’Malley Y, Fink BD, Ross NC, Prisinzano TE, Sivitz WI (2006). Reactive oxygen and targeted antioxidant administration in endothelial cell mitochondria. J. Biol. Chem..

[66] James AM, Cocheme HM, Smith RA, Murphy MP (2005). Interactions of mitochondria-targeted and untargeted ubiquinones with the mitochondrial respiratory chain and reactive oxygen species. Implications for the use of exogenous ubiquinones as therapies and experimental tools. J. Biol. Chem..

[67] James AM (2007). Interaction of the mitochondria-targeted antioxidant MitoQ with phospholipid bilayers and ubiquinone oxidoreductases. J. Biol. Chem..

[68] Zhong X (2022). Complex metabolic interactions between ovary, plasma, urine, and hair in ovarian cancer. Front. Oncol..

[69] Xiao L (2017). The mitochondria-targeted antioxidant MitoQ ameliorated tubular injury mediated by mitophagy in diabetic kidney disease via Nrf2/PINK1. Redox Biol..

[70] Dou SD (2021). MitoQ inhibits hepatic stellate cell activation and liver fibrosis by enhancing PINK1/parkin-mediated mitophagy. Open Med. (Wars).

[71] Ji Y (2022). The mitochondria-targeted antioxidant MitoQ ameliorates myocardial ischemia-reperfusion injury by enhancing PINK1/Parkin-mediated mitophagy in type 2 diabetic rats. Cell Stress Chaperones.

[72] Meka DP (2015). Parkin cooperates with GDNF/RET signaling to prevent dopaminergic neuron degeneration. J. Clin. Invest..

[73] Drisko JA, Chapman J, Hunter VJ (2003). The use of antioxidants with first-line chemotherapy in two cases of ovarian cancer. J. Am. Coll. Nutr..

[74] Pin F, Huot JR, Bonetto A (2022). The mitochondria-targeting agent MitoQ improves muscle atrophy, weakness and oxidative metabolism in C26 tumor-bearing mice. Front. Cell Dev. Biol..

[75] Wu PK (2013). A mortalin/HSPA9-mediated switch in tumor-suppressive signaling of Raf/MEK/extracellular signal-regulated kinase. Mol. Cell Biol..

[76] Hong SK, Yoon S, Moelling C, Arthan D, Park JI (2009). Noncatalytic function of ERK1/2 can promote Raf/MEK/ERK-mediated growth arrest signaling. J. Biol. Chem..

[77] Eisenhauer EA (2009). New response evaluation criteria in solid tumours: revised RECIST guideline (version 1.1). Eur. J. Cancer.

[78] U.S.-Department-of-Health-and-Human-Services. Common Terminology Criteria for Adverse Events (CTCAE) Version 4.03. (2010).

