# Supplementary material for: Selpercatinib combination with the mitochondria-targeted antioxidant MitoQ effectively suppresses RET–mutant thyroid cancer
Source: NPJ Precis Oncol. 2024 Feb 20;8:39. doi: 10.1038/s41698-024-00536-7 (PMC10879150; doi:10.1038/s41698-024-00536-7)
Supplement: Supplementary file 1 — Reporting Summary [file 41698_2024_536_MOESM1_ESM.pdf]

nature portfolio

Corresponding author(s): Jong-In Park

Last updated by author(s): Nov 20, 2023

Reporting Summary

Nature Portfolio wishes to improve the reproducibility of the work that we publish. This form provides structure for consistency and transparency in reporting. For further information on Nature Portfolio policies, see our Editorial Policies and the Editorial Policy Checklist.

Statistics

For all statistical analyses, confirm that the following items are present in the figure legend, table legend, main text, or Methods section.

n/a	Confirmed	
☐	☒	The exact sample size (*n*) for each experimental group/condition, given as a discrete number and unit of measurement
☐	☒	A statement on whether measurements were taken from distinct samples or whether the same sample was measured repeatedly
☐	☒	The statistical test(s) used AND whether they are one- or two-sided<br>*Only common tests should be described solely by name; describe more complex techniques in the Methods section.*
☐	☒	A description of all covariates tested
☐	☒	A description of any assumptions or corrections, such as tests of normality and adjustment for multiple comparisons
☐	☒	A full description of the statistical parameters including central tendency (e.g. means) or other basic estimates (e.g. regression coefficient) AND variation (e.g. standard deviation) or associated estimates of uncertainty (e.g. confidence intervals)
☒	☐	For null hypothesis testing, the test statistic (e.g. *F*, *t*, *r*) with confidence intervals, effect sizes, degrees of freedom and *P* value noted<br>*Give P values as exact values whenever suitable.*
☒	☐	For Bayesian analysis, information on the choice of priors and Markov chain Monte Carlo settings
☒	☐	For hierarchical and complex designs, identification of the appropriate level for tests and full reporting of outcomes
☒	☐	Estimates of effect sizes (e.g. Cohen's *d*, Pearson's *r*), indicating how they were calculated

Our web collection on statistics for biologists contains articles on many of the points above.

Software and code

Policy information about availability of computer code

Data collection	N/A
Data analysis	N/A

For manuscripts utilizing custom algorithms or software that are central to the research but not yet described in published literature, software must be made available to editors and reviewers. We strongly encourage code deposition in a community repository (e.g. GitHub). See the Nature Portfolio guidelines for submitting code & software for further information.

Data

Policy information about availability of data

All manuscripts must include a data availability statement. This statement should provide the following information, where applicable:

- Accession codes, unique identifiers, or web links for publicly available datasets
- A description of any restrictions on data availability
- For clinical datasets or third party data, please ensure that the statement adheres to our policy

All data needed to evaluate the conclusions in the paper are present in the paper and the Supplementary Materials.

Research involving human participants, their data, or biological material

Policy information about studies with human participants or human data. See also policy information about sex, gender (identity/presentation), and sexual orientation and race, ethnicity and racism.

Reporting on sex and gender	The first patient is male. The second patient is female.
Reporting on race, ethnicity, or other socially relevant groupings	*Please specify the socially constructed or socially relevant categorization variable(s) used in your manuscript and explain why they were used. Please note that such variables should not be used as proxies for other socially constructed/relevant variables (for example, race or ethnicity should not be used as a proxy for socioeconomic status).*<br>*Provide clear definitions of the relevant terms used, how they were provided (by the participants/respondents, the researchers, or third parties), and the method(s) used to classify people into the different categories (e.g. self-report, census or administrative data, social media data, etc.)*<br>*Please provide details about how you controlled for confounding variables in your analyses.*
Population characteristics	*Describe the covariate-relevant population characteristics of the human research participants (e.g. age, genotypic information, past and current diagnosis and treatment categories). If you filled out the behavioural & social sciences study design questions and have nothing to add here, write "See above."*
Recruitment	*Describe how participants were recruited. Outline any potential self-selection bias or other biases that may be present and how these are likely to impact results.*
Ethics oversight	The patients were treated at the Froedtert Hospital and Medical College of Wisconsin. Analysis and reporting of patients' treatment and outcome was performed under an Institutional Review Board approved study (IRB number PRO00002787) that waives the requirement for informed consent because of the use of de-identified data.

Note that full information on the approval of the study protocol must also be provided in the manuscript.

Field-specific reporting

Please select the one below that is the best fit for your research. If you are not sure, read the appropriate sections before making your selection.

☒ Life sciences ☐ Behavioural & social sciences ☐ Ecological, evolutionary & environmental sciences

For a reference copy of the document with all sections, see nature.com/documents/nr-reporting-summary-flat.pdf

Life sciences study design

All studies must disclose on these points even when the disclosure is negative.

Sample size	For in vitro experiments, at least three biological replicates were used For in vivo studies, samples size were determined by consulting with a biostatistician.
Data exclusions	3 mice were excluded, because they had to be sacrificed due to an injury caused by oral gavage.
Replication	All experiments were replicated.
Randomization	When the tumor volume reached 50 mm3, mice were randomized and sorted to achieve equal tumor size distribution across the groups.
Blinding	*Describe whether the investigators were blinded to group allocation during data collection and/or analysis. If blinding was not possible, describe why OR explain why blinding was not relevant to your study.*

Reporting for specific materials, systems and methods

We require information from authors about some types of materials, experimental systems and methods used in many studies. Here, indicate whether each material, system or method listed is relevant to your study. If you are not sure if a list item applies to your research, read the appropriate section before selecting a response.

Materials & experimental systems

n/a	Involved in the study
☐	☒ Antibodies
☐	☒ Eukaryotic cell lines
☒	☐ Palaeontology and archaeology
☐	☒ Animals and other organisms
☐	☒ Clinical data
☒	☐ Dual use research of concern
☒	☐ Plants

Methods

n/a	Involved in the study
☒	☐ ChIP-seq
☐	☒ Flow cytometry
☒	☐ MRI-based neuroimaging

Antibodies

Antibodies used

RET (Cell Signaling, Danvers, MA, #3223); phospho-RET Try905 (Cell Signaling, #3221); ERK (Cell Signaling, #9102); phospho-ERK Thr202/Tyr204 (Santa Cruz Biotechnology, Santa Cruz, CA, #sc-16982); AKT (Cell Signaling, #9272); phospho-AKT Ser473 (Cell Signaling, #9271); PARP (Cell Signaling, #9542); cleaved lamin A (Cell Signaling, #2035); cytochrome c oxidase subunit IV (COX IV, Cell Signaling, #4850); p21CIP1 (Santa Cruz Biotechnology, #sc-56335); p27KIP1 (Santa Cruz Biotechnology, #sc-1641); HIF1α (Cell Signaling, #14179) and β-actin (MilliporeSigma, #A2228).

Validation

All antibodies listed above were validated by manufacturers.

Eukaryotic cell lines

Policy information about cell lines and Sex and Gender in Research

Cell line source(s)

TPC1 cell line was derived from papillary thyroid carcinoma from adult Japanese female; TT cell line was derived from medullary thyroid carcinoma from a 77 years old Caucasian female; MZ-CRC-1 cell line was derived from medullary thyroid carcinoma from a 43 years old female. PCCL3 cell line was derived from rat thyroid gland; HEK293 cell was derived from human fetal kidney.

Authentication

Cell lines were authenticated in previous studies

Mycoplasma contamination

Cells were not contaminated

Commonly misidentified lines
(See ICLAC register)

Name any commonly misidentified cell lines used in the study and provide a rationale for their use.

Animals and other research organisms

Policy information about studies involving animals; ARRIVE guidelines recommended for reporting animal research, and Sex and Gender in Research

Laboratory animals

Female athymic nude (nu/nu) mice (The Jackson Laboratory, Bar Harbour, ME, #007850)

Wild animals

No

Reporting on sex

Female

Field-collected samples

No

Ethics oversight

All animal studies were performed according to the protocol approved by the Institutional Animal Care and Use Committee at the Medical College of Wisconsin (AUA00001327).

Note that full information on the approval of the study protocol must also be provided in the manuscript.

Clinical data

Policy information about clinical studies

All manuscripts should comply with the ICMJE guidelines for publication of clinical research and a completed CONSORT checklist must be included with all submissions.

Clinical trial registration

Provide the trial registration number from ClinicalTrials.gov or an equivalent agency.

Study protocol

Note where the full trial protocol can be accessed OR if not available, explain why.

Data collection

Describe the settings and locales of data collection, noting the time periods of recruitment and data collection.

Outcomes

Describe how you pre-defined primary and secondary outcome measures and how you assessed these measures.

Plants

Seed stocks

N/A

Novel plant genotypes

Describe the methods by which all novel plant genotypes were produced. This includes those generated by transgenic approaches, gene editing, chemical/radiation-based mutagenesis and hybridization. For transgenic lines, describe the transformation method, the number of independent lines analyzed and the generation upon which experiments were performed. For gene-edited lines, describe the editor used, the endogenous sequence targeted for editing, the targeting guide RNA sequence (if applicable) and how the editor was applied.

Authentication

Describe any authentication procedures for each seed stock used or novel genotype generated. Describe any experiments used to assess the effect of a mutation and, where applicable, how potential secondary effects (e.g. second site T-DNA insertions, mosiacism, off-target gene editing) were examined.

Flow Cytometry

Plots

Confirm that:

☒ The axis labels state the marker and fluorochrome used (e.g. CD4-FITC).

☒ The axis scales are clearly visible. Include numbers along axes only for bottom left plot of group (a 'group' is an analysis of identical markers).

☒ All plots are contour plots with outliers or pseudocolor plots.

☒ A numerical value for number of cells or percentage (with statistics) is provided.

Methodology

Sample preparation

Please see method section for cell viability, cell cycle analyses, and detection of mitochondria membrane potential sections.

Instrument

Guava EasyCyte flow cytometry system (MilliporeSigma, #05005007)

Software

FCS EXPRESS software (De Novo Software, Los Angeles, California)

Cell population abundance

Cells were not sorted.

Gating strategy

FSC/SSC gating is about 60% of total population. Following gating on positive/negative are determined by negative control (non-stained, or no treated), usually <10^2 is considered negative, whereas >10^2 is positive.

☐ Tick this box to confirm that a figure exemplifying the gating strategy is provided in the Supplementary Information.
