# Supplementary material for: Selpercatinib combination with the mitochondria-targeted antioxidant MitoQ effectively suppresses RET–mutant thyroid cancer
Source: NPJ Precis Oncol. 2024 Feb 20;8:39. doi: 10.1038/s41698-024-00536-7 (PMC10879150; doi:10.1038/s41698-024-00536-7)
Supplement: Supplementary file 2 — Supplementary figures and patient case info [file 41698_2024_536_MOESM2_ESM.pdf]

Supplementary Figure 1.

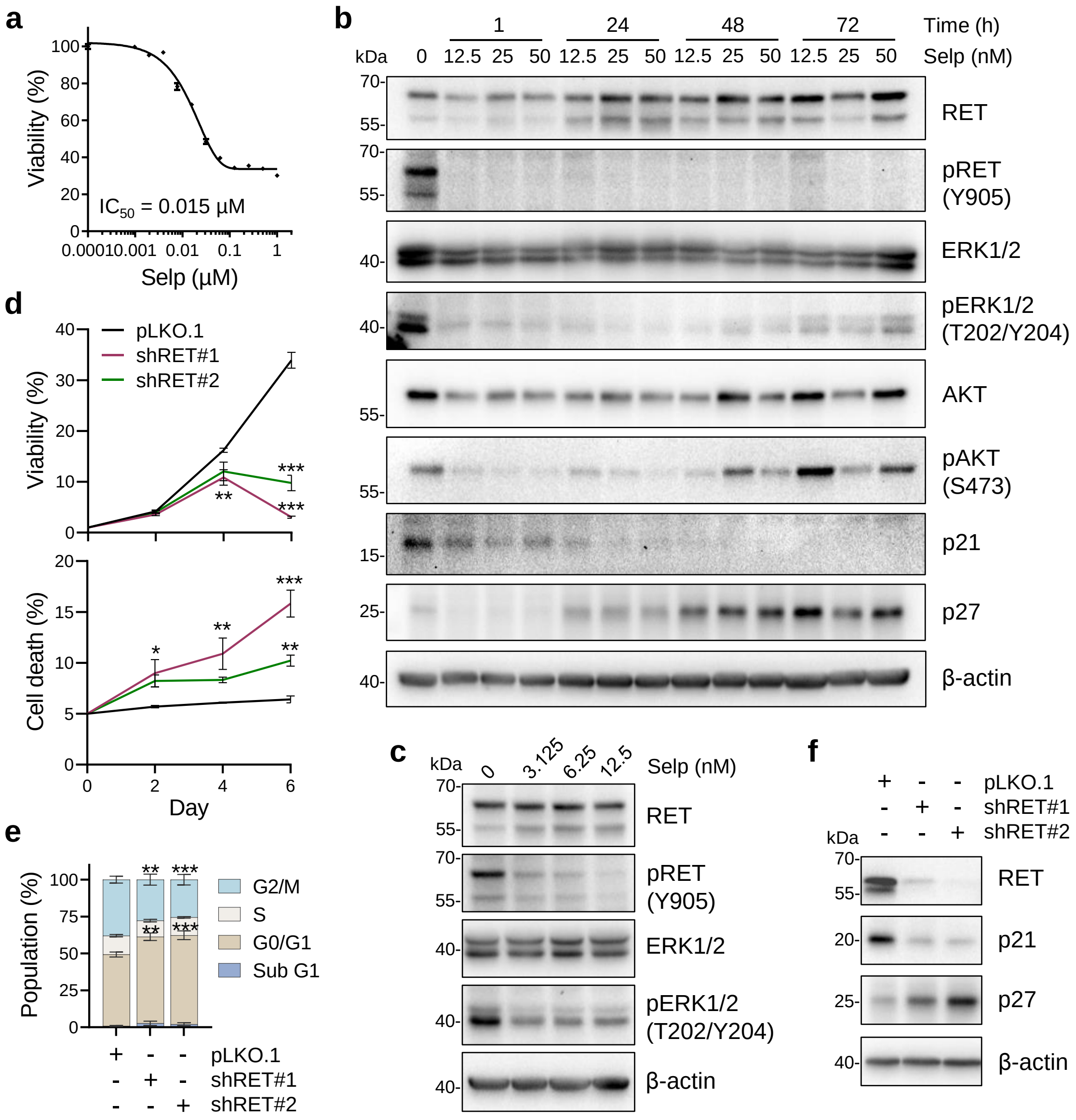

Selpercatinib and RNA interference of RET consistently suppress the viability of TPC1 cells. (a) Cells were treated with increasing doses of selpercatinib (Selp) in 12 well plates for 72 hours prior to crystal violet viability assay. Data (mean ± SEM, N = 3) are expressed as the percentage of untreated controls. **(b)** Time-course Western blot analysis of total lysates of cells treated with different doses of selpercatinib. β-actin is the control for equal protein loading. **(c)** Western blot analysis of total lysates of cells treated with lower doses of selpercatinib for 48 hours. **(d)** TO-PRO-3 assay (mean ± SEM, N = 3) of cells infected with lentiviral pLKO.1-shRET#1 and shRET#2. *P < 0.05, **P < 0.005, ***P < 0.001, Two-Way ANOVA with Bonferroni post-tests. **(e)** Cell cycle analysis (mean ± SEM, N = 3) of cells infected with lentiviral pLKO.1-shRET#1 and shRET#2. **P < 0.005, ***P < 0.001, One-Way ANOVA with Bonferroni post-tests. **(f)** Representative images for Western Blot analysis of cells in (d and e).

Supplementary Figure 2.

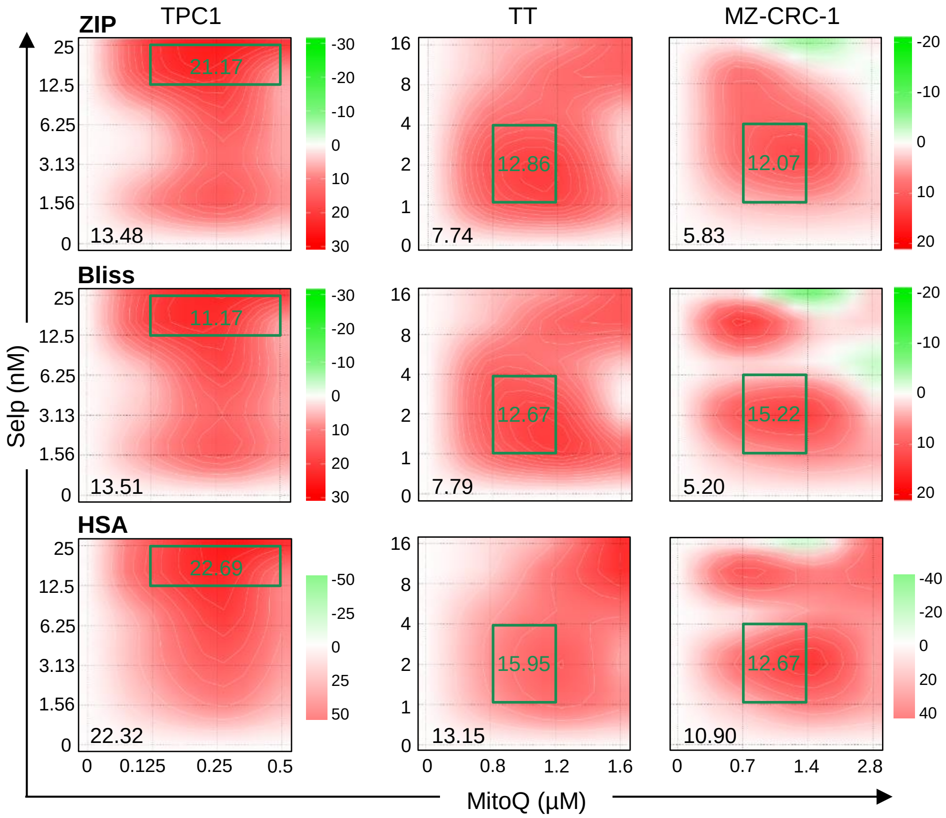

SynergyFinder analyses of the viability data in Figure 2c and 2j. The synergy effects of selpercatinib and MitoQ in TPC1, TT, and MZ-CRC-1 cell lines were analyzed using ZIP, Bliss, and HSA. Numbers in squares and on bottom left indicate the scores for most synergistic area and the overall synergy scores, respectively.

Supplementary Figure 3.

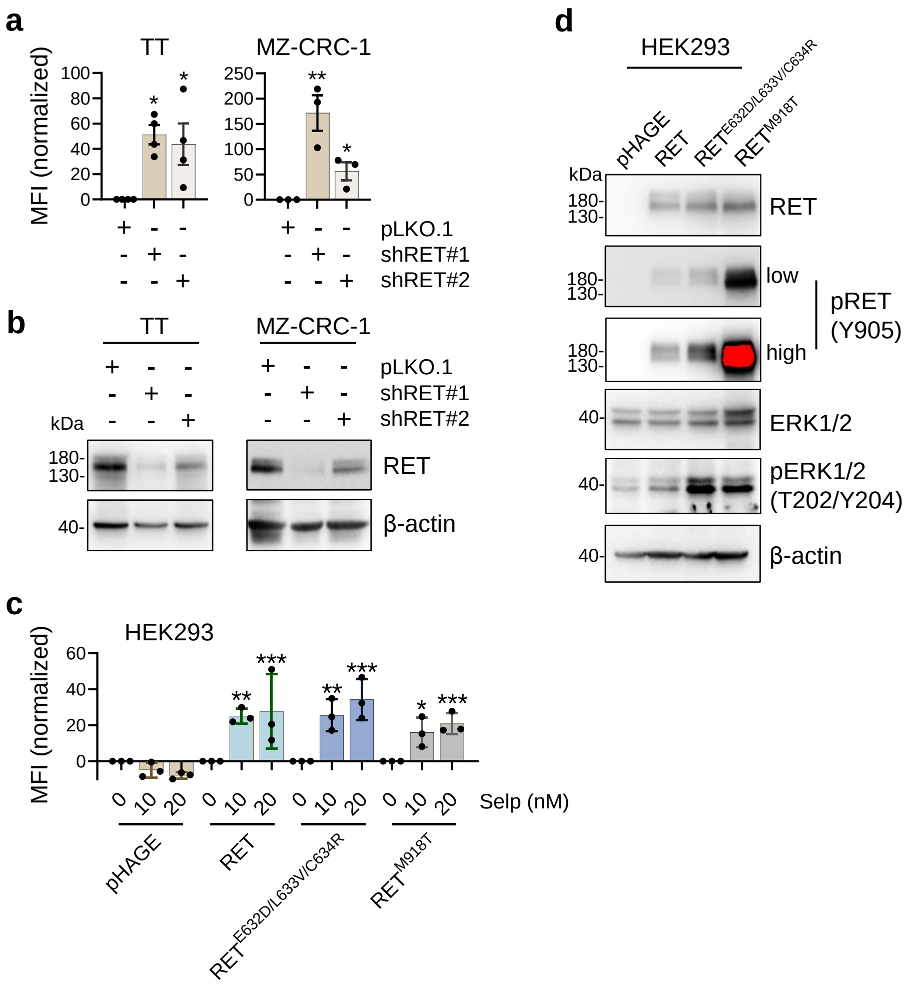

RET inhibition or depletion increases mitochondrial membrane potential in cells with increased RET activity. (a) MFI of TMRM-stained TT ($N = 4$) and MZ-CRC-1 ($N = 3$) cells infected with lentiviral pLKO.1-shRET#1 and shRET#2 for 2 days. Signals were quantified by FCS Express software. Data are mean ± SEM. *$P < 0.05$, **$P < 0.005$ relative to pLKO.1, One-Way ANOVA with Bonferroni post-tests. **(b)** Western blot analysis of total lysates of cells described in (a). **(c)** MFI of TMRM-stained HEK293 cells infected with lentiviral pHAGE-*RET*, *RET*^{C634W} and *RET*^{M918T}. Cells were treated with different dosages of selpercatinib for 48 hours before the assay. Data are mean ± SEM ($N = 3$). *$P < 0.05$, **$P < 0.005$, ***$P < 0.001$ relative to untreated, One-Way ANOVA with Bonferroni post-tests. **(d)** Western blot analysis of total lysates of cells infected with lentiviral pHAGE-*RET*, *RET*$^{E632D/L633V/C634R}$ and *RET*^{M918T}.

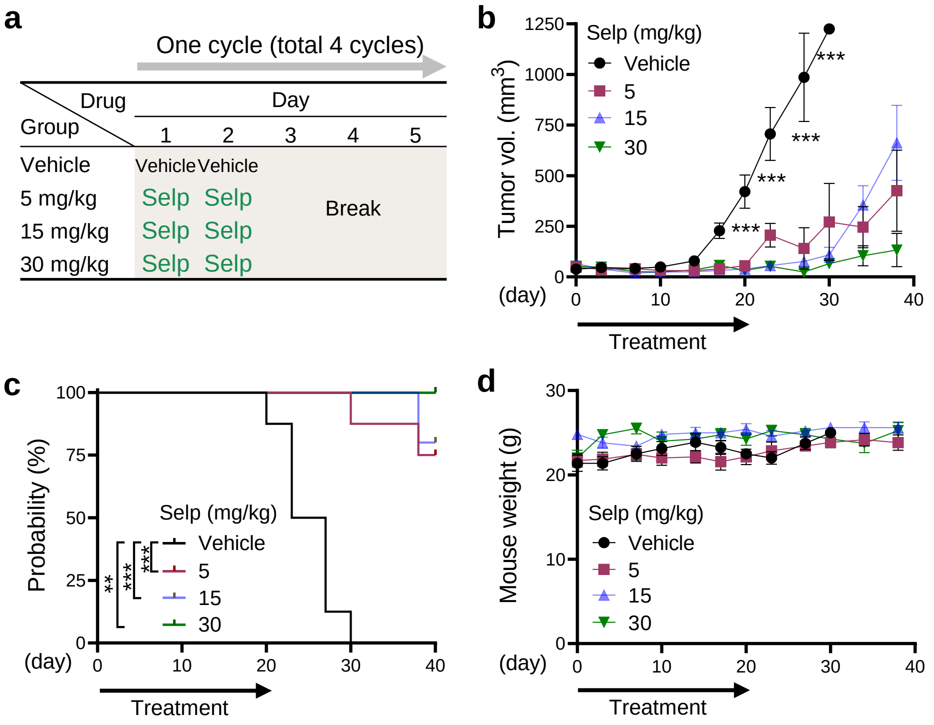

Selpercatinib dose determination in athymic nude mice bearing TPC1 xenografts. (a) Treatment schedule. Selpercatinib dissolved in 100 μL vehicle (1:12 mixture of DMSO/15% β-cyclodextrin) were orally administered by gavage. **(b)** Changes in tumor sizes. Vehicle group (n = 8), 5 mg/kg group (n = 7), 15 mg/kg group (n = 5), 30 mg/kg group (n = 4). ***$p < 0.001$ relative to vehicle, Two-way ANOVA with Bonferroni post-tests. **(c)** Survival probabilities were plotted with the tumor size over 1200 mm^3 as the endpoint. *$p < 0.05$, **$p < 0.005$, ***$p < 0.001$, Log-rank test. **(d)** Body weights of animals measured at the end of the experiment.

Supplementary Figure 5.

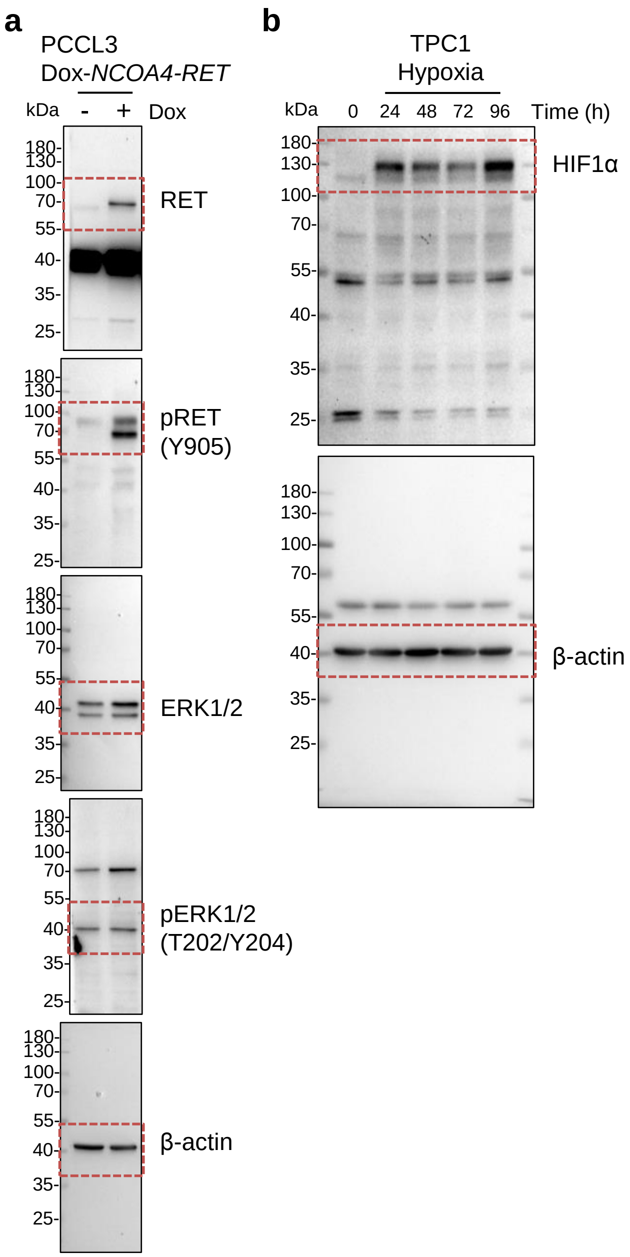

c

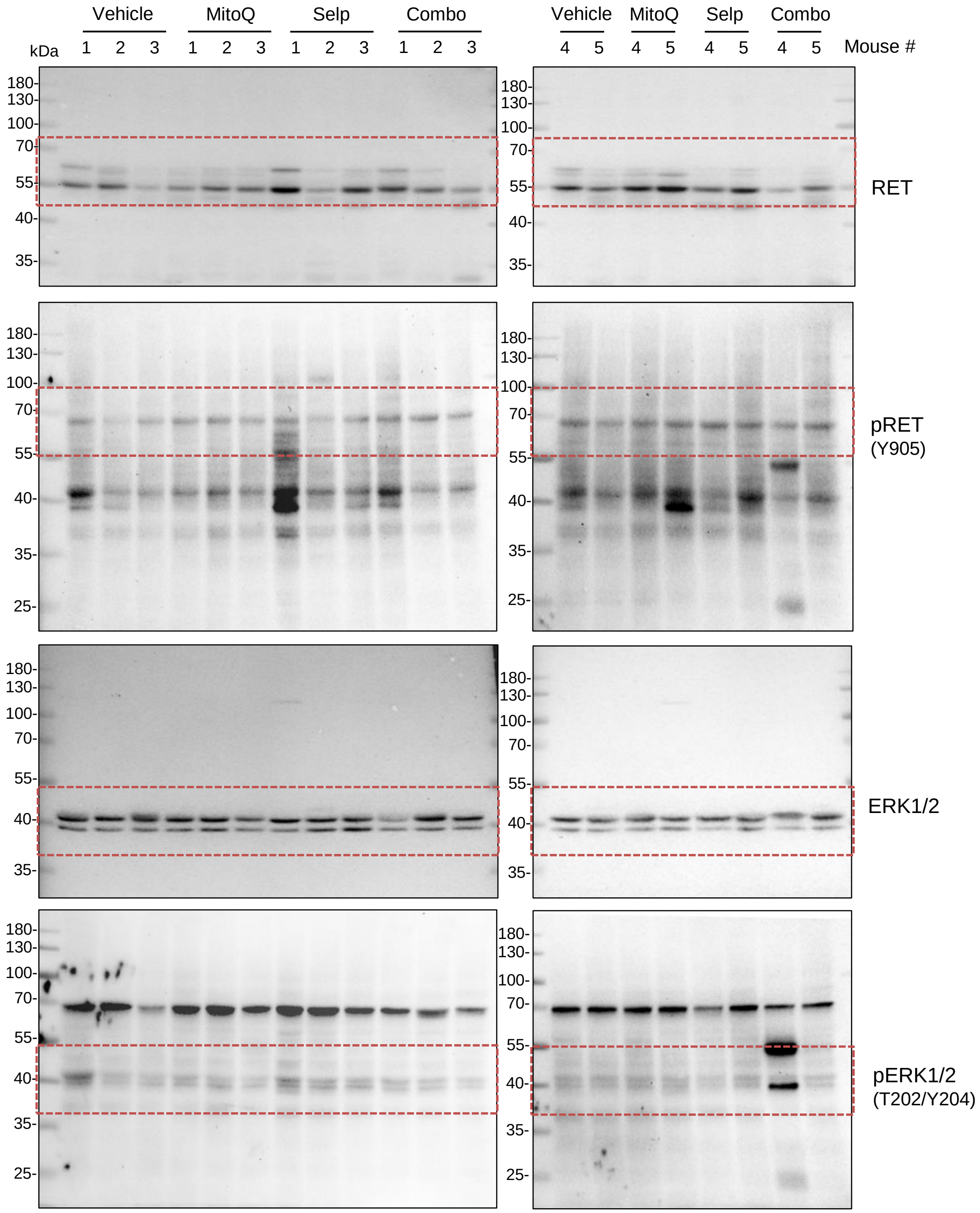
Vehicle
MitoQ
Selp
Combo
kDa
1 2 3 1 2 3 1 2 3 1 2 3
Vehicle
MitoQ
Selp
Combo
4 5 4 5 4 5 4 5
Mouse #
180-
130-
100-
70-
55-
40-
35-
25-
RET
pRET
(Y905)
ERK1/2
pERK1/2
(T202/Y204)

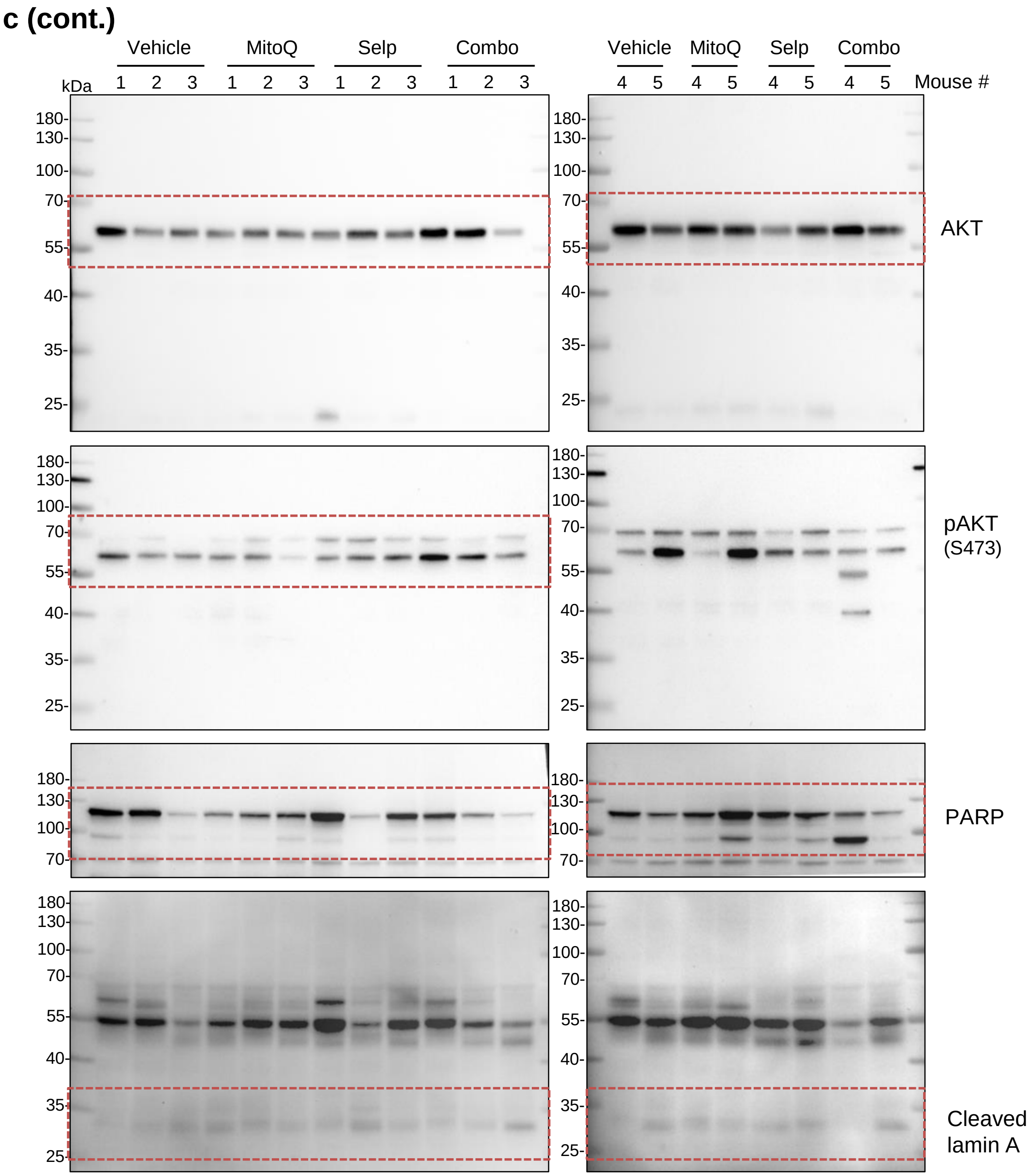
c (cont.)
Vehicle
MitoQ
Selp
Combo
1 2 3 1 2 3 1 2 3 1 2 3
Vehicle
MitoQ
Selp
Combo
4 5 4 5 4 5 4 5
Mouse #
kDa
180-
130-
100-
70-
55-
40-
35-
25-
AKT
pAKT
(S473)
PARP
Cleaved
lamin A

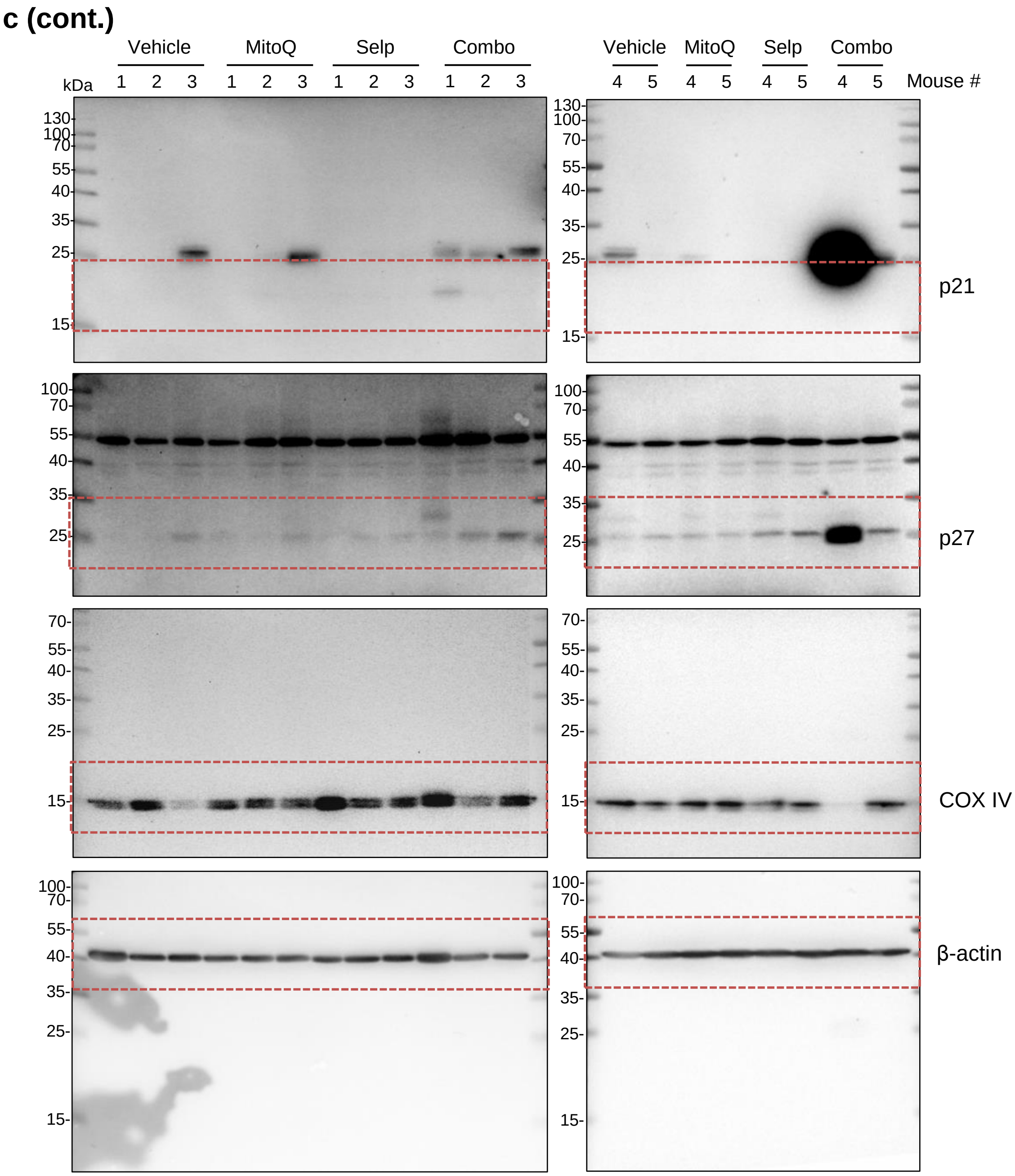
c (cont.)
Vehicle
MitoQ
Selp
Combo
kDa
1 2 3 1 2 3 1 2 3 1 2 3
4 5 4 5 4 5 4 5
Mouse #
130-
100-
70-
55-
40-
35-
25-
15-
p21
p27
COX IV
β-actin

Supplementary Figure 5. (cont.)

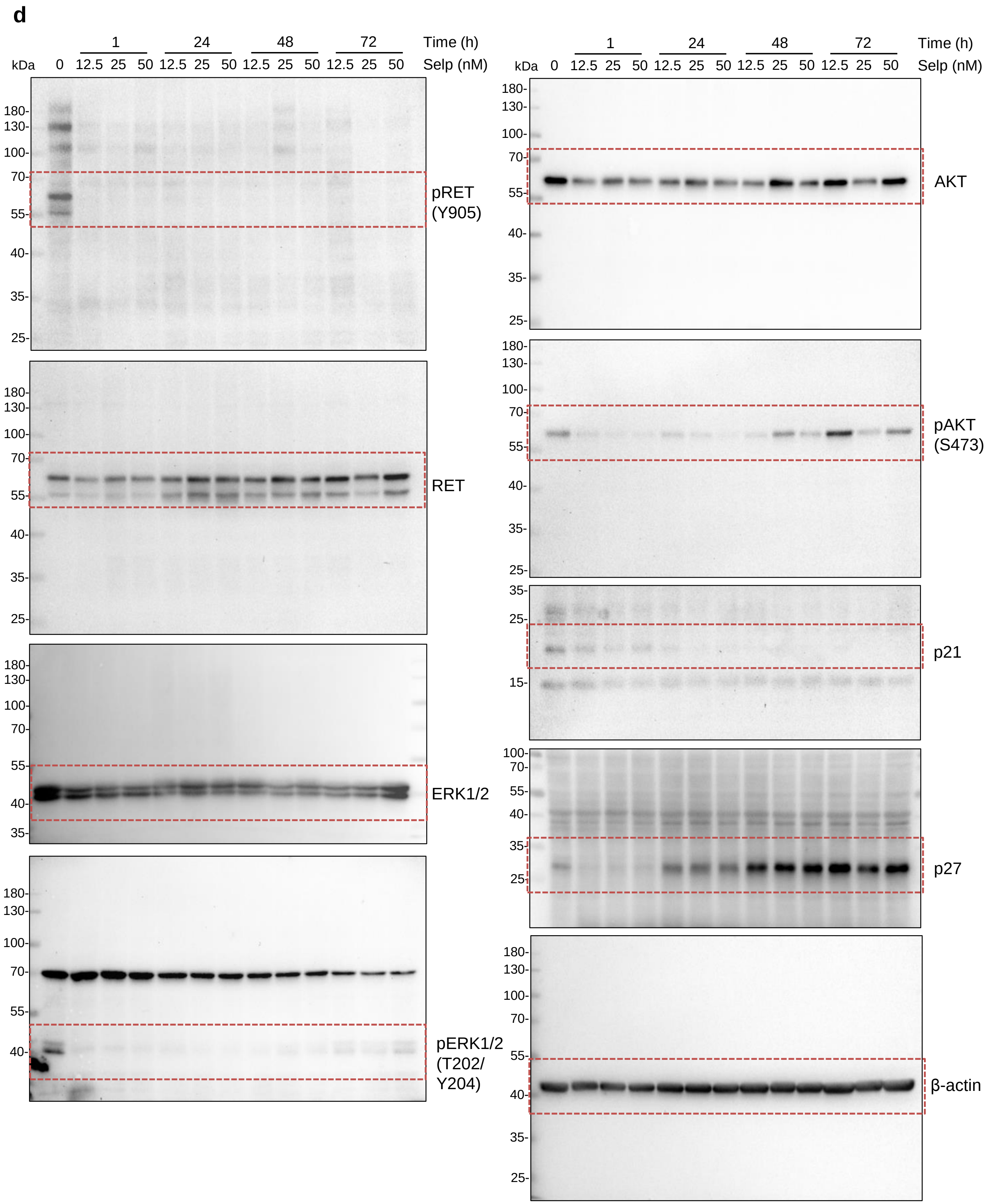

Supplementary Figure 5.

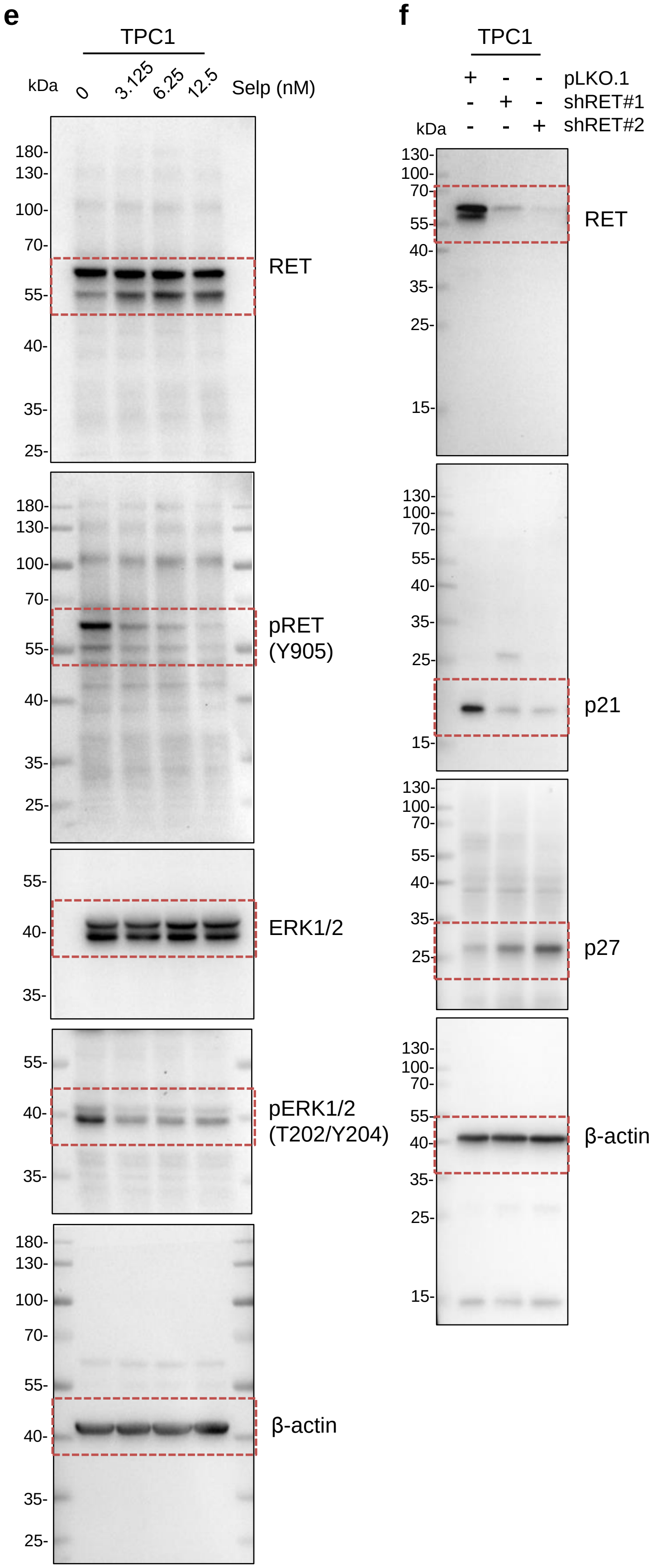

Supplementary Figure 5. (cont.)

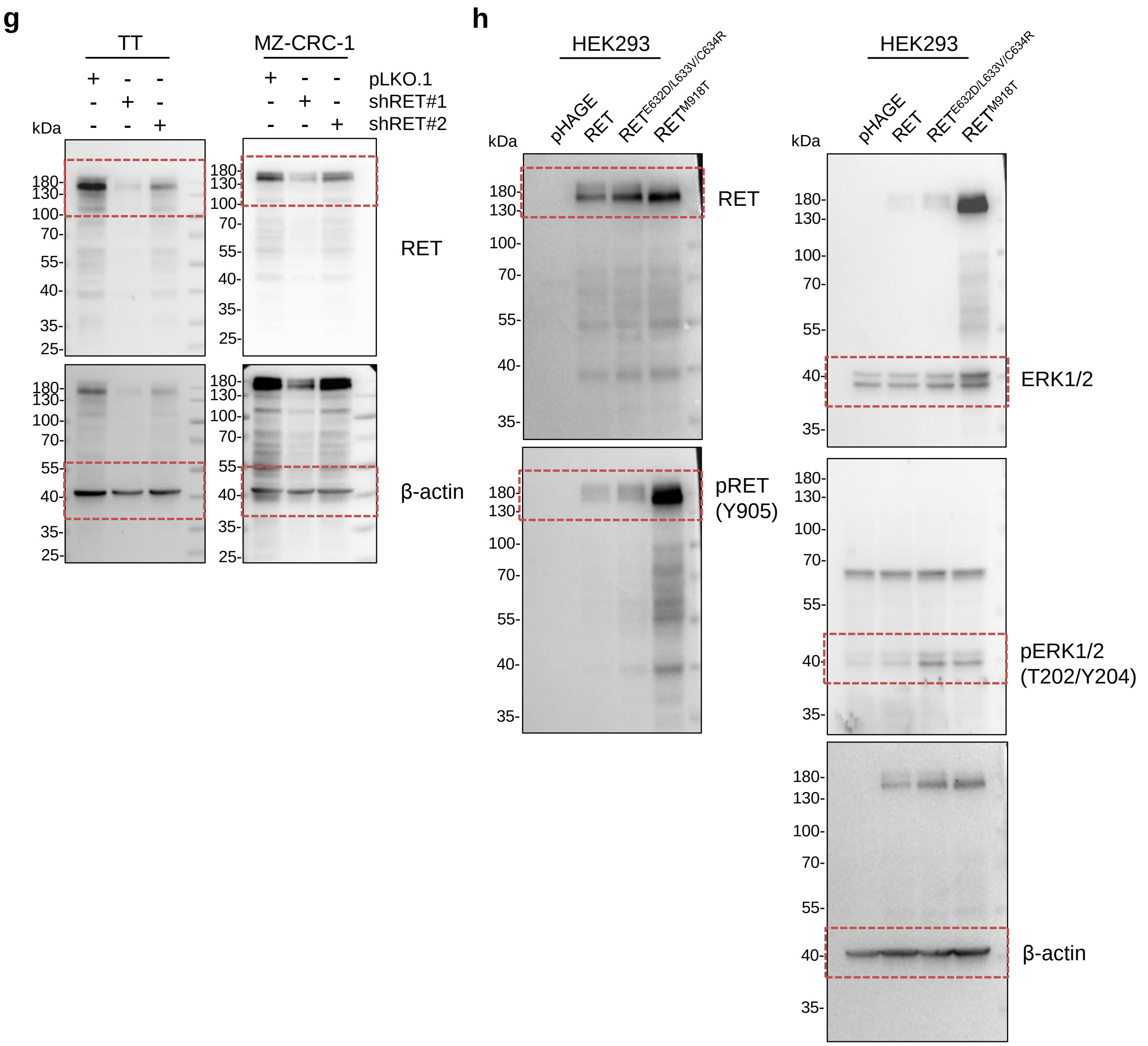

Original uncropped full blot images for the Western blotting data. (a) Images for Fig. 1f. **(b)** Images for Fig. 2f. **(c)** Images for Fig. 3e. **(d)** Images for Supplementary Fig. 1b. **(e)** Images for Supplementary Fig. 1c. **(f)** Images for Supplementary Fig. 1f. **(g)** Images for Supplementary Fig. 3b. **(h)** Images for Supplementary Fig. 3d.

Case 1:

The first case is a 55-year-old male with a history of poorly controlled hypertension who presented to the emergency department with flank pain. The patient was found to have a kidney stone, and the passage of the stone resolved his pain. While in the emergency room, a computerized tomography (CT) scan of the abdomen/pelvis revealed multiple lung nodules and a 1.7 cm lesion in the right liver, concerning for metastatic disease. The patient reported complaints of voice changes, intermittent dysphagia, and multiple neck masses. He was referred for outpatient work-up and treatment. CT scans of the neck and chest was performed and demonstrated a large right thyroid mass with right central, lateral, and mediastinal lymphadenopathy; the thyroid tumor had significant contact with the junction of the cricoid and thyroid cartilage without intralaryngeal or intratracheal extension of the tumor. There were also diffuse pulmonary nodules and small hepatic and adrenal lesions concerning for metastatic foci. The patient underwent fine needle aspiration of the right thyroid mass and suspicious right level 4 cervical lymph node. Cytology report demonstrated findings consistent with thyroid carcinoma (Bethesda VI) with atypical large cells with irregular eccentric nuclei, prominent nucleoli, and ample cytoplasm with morphology that did not fit typical papillary, follicular, or medullary carcinoma consistent with poorly differentiated thyroid carcinoma. Immunohistochemistry staining was positive for TTF-1, PAX8, and thyroglobulin, but testing was negative for calcitonin, synaptophysin, and chromogranin. Laryngoscopy showed decreased right vocal fold mobility consistent with right recurrent laryngeal nerve involvement. The patient underwent total thyroidectomy with bilateral central neck dissection, right modified radical neck dissection and removal of the substernal mass. Surgical pathology demonstrated 6.8 x 6.1 x 3.8 cm^3 multifocal bilateral papillary carcinoma, a diffuse sclerosing variant with vascular invasion, and 3 of 17 lymph nodes with metastatic disease from levels I through V. The patient was started on levothyroxine postoperatively and was treated with 153.8 mCi of I-131 4 weeks postoperatively.

After completion of I-131 treatment, a chest CT-guided biopsy of a left upper lobe lesion was performed due to concern for disease progression. Pathology from the lung lesion was consistent with poorly differentiated thyroid cancer. A magnetic resonance imaging (MRI) of the brain did not demonstrate brain metastasis. While awaiting approval for next-generation sequencing (NGS), the patient was started on lenvatinib 20 mg daily (QD). After 5 weeks of the treatment, lenvatinib was held for 4 weeks due to exacerbation of refractory hypertension to grade 3 with bilateral lower extremity edema. During this time, the patient continued worsening his hypertension despite an escalation of medical management and worsened extremity edema and cough consistent with congestive heart failure (CHF) with preserved ejection fraction (EF). NGS testing was performed of the tumor and was negative for BRAF mutation but did demonstrate *RET* mutation (*CCDC6-RET* fusion; *CDKN2B* loss; *CDKN2A* loss; *TERT* promoter$^{-124C>T}$; *TP53*^{R248Q}). While lenvatinib was held, the patient continued to have the progression of his poorly differentiated thyroid cancer. The patient's refractory hypertension and CHF with preserved EF persisted despite being off lenvatinib. Concern was raised regarding his deteriorating medical status and fear that initiation of selpercatinib at standard dose would limit the effectiveness of his anti-cancer therapy. After a multidisciplinary discussion, the decision was made to initiate a reduced dose schedule with the addition of MitoQ, possibly potentiating the effect of selpercatinib, which could be safer from a cardiovascular standpoint while potentially avoiding the need for therapy interruption. The patient and his wife were counseled regarding the necessity to initiate treatment in a manner that could control his cancer but would limit cardiac toxicity in the setting where there was no safe and effective standard of treatment. A treatment with selpercatinib and MitoQ was started on a weekly cycled schedule: selpercatinib 160 mg twice a day (BID) on days 1, 2 (off days 3, 4, 5, 6, 7) followed by MitoQ 20 mg QD on days 3, 4, 5, 6, 7 (off days 1, 2).

The patient remained on this dose of selpercatinib and MitoQ for 22 months, with a near-complete response following the first four months of treatment. At 23 months of treatment, CT

scans of the neck and chest demonstrated a new site of oligometastatic disease in the manubrium, which progressed over the next month. At this time, MitoQ was stopped, and the selpercatinib dose was escalated to 160mg BID with plans for additional treatment with external beam radiation.

Case 2:

The second case is a 34-year-old female with a right lateral neck mass that she had for about six months and grew slowly but steadily over that period before the presentation. She sought care when she began having additional symptoms of diarrhea, with the mass becoming firm and painful. The patient underwent a thyroid ultrasound, which revealed a right thyroid mass with extracapsular extension and multiple suspicious right lateral neck lymph nodes. Fine needle aspiration of her thyroid mass and right level 2 lymph node were performed and demonstrated medullary thyroid cancer. Carcinoembryonic antigen (CEA) and calcitonin levels were both elevated at 231.1 (CEA reference range ≤ 4.7) and 8196 pg/mL (Calcitonin reference range 0-5 pg/mL), respectively. The patient was started on loperamide and lanreotide for her diarrhea which improved after starting these medications. CT scan of the neck, chest, abdomen, and pelvis demonstrated a 7 cm mass of the right thyroid involving the isthmus creating a mass effect on the carotid sheath, trachea, and esophagus with pathologic adenopathy in right levels II through IV and bilateral level VI nodes. Imaging was concerning for encasement of the right common carotid with the trachea and esophagus involvement. MRI of the brain was also performed because of a new onset of headaches and right earache, but it did not demonstrate metastatic disease in the brain. Due to symptoms of hoarseness of voice and dysphagia, a laryngoscopy was performed and demonstrated mass effect but normal vocal fold movement. A multidisciplinary decision to pursue immunotherapy was made because complete resection was impossible without significant morbidity. Repeat fine needle aspiration was performed, and NGS testing of the tissue identified a *RET* mutation (p.M918T missense variant-GOF). The patient was started on selpercatinib 160 mg BID. When she was continued on it for seven weeks, she had a grade 4, asymptomatic alanine transferase (ALT) and aspartate transferase (AST) elevation. At that time, selpercatinib was held due to hepatotoxicity, and CT scans of the neck and chest demonstrated a slight decrease in the size of the right thyroid mass with less mass effect on the trachea and cervical esophagus, slightly less mass effect on the right carotid sheath and minimal decrease in right level 2A and level 3 adenopathy. After the liver function test normalized, selpercatinib was resumed at 80 mg BID (dose level -2 in accordance with FDA approved package insert), but she developed recurrent grade 2 transaminase elevation. After resolution of the liver function tests, she resumed selpercatinib adding MitoQ using the following weekly cycled schedule: selpercatinib 80mg BID on days 1, 2 (off days 3-7) followed by MitoQ 10 mg QD on days 3-7 (off days 1 and 2) to potentiate the effect of selpercatinib while diminishing the hepatotoxic side effects. Follow-up imaging after seven weeks on this regimen was consistent with reduced tumor size and treatment response. This treatment regimen was continued with a plateau of response on imaging at 24 weeks of treatment. After a multidisciplinary discussion, she underwent a right thyroidectomy, right level VI and VII lymph node dissection, and right recurrent laryngeal nerve resection. Surgical pathology demonstrated 4.1 cm medullary thyroid cancer with lymphovascular invasion, perineural invasion, extensive extrathyroidal extension, and 16 of 27 right level I-V lymph nodes positive for metastatic medullary thyroid cancer with extranodal extension and perineural invasion. She is currently undergoing treatment with adjuvant external beam radiation.
